# Gaining insight into the assimilated diet of small bear populations by stable isotope analysis

**DOI:** 10.1038/s41598-021-93507-y

**Published:** 2021-07-08

**Authors:** Giulio Careddu, Paolo Ciucci, Stella Mondovì, Edoardo Calizza, Loreto Rossi, Maria Letizia Costantini

**Affiliations:** 1 Department of Environmental Biology, Sapienza University of Rome, Rome, Italy; 2 Department of Biology and Biotechnologies “Charles Darwin”, Sapienza University of Rome, Rome, Italy

**Keywords:** Conservation biology, Ecology, Stable isotope analysis, Conservation biology, Ecology, Stable isotope analysis

## Abstract

Apennine brown bears (*Ursus arctos marsicanus*) survive in an isolated and critically endangered population, and their food habits have been studied using traditional scat analysis. To complement current dietary knowledge, we applied Stable Isotope Analysis (SIA) to non-invasively collected bear hairs that had been individually recognized through multilocus genotyping. We analysed carbon (δ^13^C) and nitrogen (δ^15^N) stable isotopes of hair sections and bear key foods in a Bayesian mixing models framework to reconstruct the assimilated diet on a seasonal basis and to assess gender and management status effects. In total, we analysed 34 different seasonal bear key foods and 35 hair samples belonging to 27 different bears (16 females and 11 males) collected during a population survey in 2014. Most bears showed wide δ^15^N and δ^13^C ranges and individual differences in seasonal isotopic patterns. Vegetable matter (herbs, fleshy fruits and hard mast) represented the major component of the assimilated diet across the dietary seasons, whereas vegetable crops were rarely and C4 plants (i.e., corn) never consumed. We confirmed an overall low consumption of large mammals by Apennine bears consistently between sexes, with highest values in spring followed by early summer but null in the other seasons. We also confirmed that consumption of fleshy fruits peaked in late summer, when wild predominated over cultivated fleshy fruits, even though the latter tended to be consumed in higher proportion in autumn. Male bears had higher δ ^15^N values than females in spring and autumn. Our findings also hint at additional differences in the assimilated diet between sexes, with females likely consuming more herbs during spring, ants during early summer, and hard mast during fall compared to males. In addition, although effect sizes were small and credibility intervals overlapped considerably, management bears on average were 0.9‰ lower in δ ^13^C and 2.9‰ higher in δ ^15^N compared to non-management bears, with differences in isotopic values between the two bear categories peaking in autumn. While non-management bears consumed more herbs, wild fleshy fruits, and hard mast, management bears tended to consume higher proportions of cultivated fruits, ants, and large mammals, possibly including livestock. Although multi-year sampling and larger sample sizes are needed to support our findings, our application confirms that SIA can effectively integrate previous knowledge and be efficiently conducted using samples non-invasively collected during population surveys.

## Introduction

Knowledge of dietary requirements of wildlife species is fundamental to understand their physiology and ecology, to assess the adequacy of food resources and their availability, and to accordingly inform management and conservation planning^1,2^. Availability, accessibility, and nutritional quality of food resources can markedly affect population dynamics^3^ and determine the extent to which wildlife species resort to anthropogenic foods, thereby generating conflicts with humans^4–6^. Bears are large-bodied and highly opportunistic omnivores whose diet comprises a great diversity of vegetable matter and a variable amount of meat and invertebrates, with marked variation according to latitude, season, and habitat productivity^7^. Depending on local conditions, brown bears may cause conflicts with humans due to their ability to exploit anthropogenic food subsidies^8–10^. Especially in human-modified landscapes, detailed knowledge of the feeding ecology of bears can efficiently guide conservation planning^11–13^, and this is important to inform both long-term habitat management^14,15^ and management of food-conditioned bears^16^. Accordingly, a consistent number of dietary studies have been carried out on several bear populations^7,8,17–21^. These revealed that dietary differences reflect different nutritional and energetic requirements of male and female bears^22,23^, differences in their life cycle, as well as spatial and habitat segregation between sexes due to behavioural mechanisms^19^.

Traditionally, food-habit studies on bears have been conducted by means of scat analysis, a rather convenient and practical technique that nevertheless does not necessarily allow an adequate interpretation in terms of assimilated diet^15,24,25^. In addition, quantification of undigested remains in the scats may largely underestimate occurrence of soft and highly digestible food items, therefore undervaluing their relative nutritional contribution^15,24,25^. Alternatively, stable isotope analysis (SIA) of carbon (C) and nitrogen (N) has been increasingly applied to bears^17,20,24,26–28^. Based on whole organisms or samples of their tissues^29–32^, SIA is based on the evidence that isotopic values are directly related to the assimilated diet, since tissues are synthetized from the nutrients formerly assimilated, reflecting their isotopic composition in a predictable manner^33,34^. More recently, Bayesian mixing models have been used to discriminate the proportional contributions of different assimilated food items to the consumer diet^28,35–37^. Concerning Ursids, SIA has clarified some important issues regarding their feeding ecology that could otherwise not be resolved with traditional scat analysis, from the paleoecology of ancient or of extinct bears populations^24,38^, to the contribution of salmon in the assimilated diet of North American brown bears^39^, or the relevance of white-bark pine nuts for grizzly bears in the Yellowstone^40,41^. SIA has been also successfully applied to detect crop foraging bears^21,42^ and bears conditioned to anthropogenic foods^20,43–45^. SIA has also been applied to small and endangered bear populations (e.g., Asiatic black bears *Ursus thibetanus*^26,27^ and Hokkaido brown bear^46,47^) but, with the sole exception of the bear population in Slovenia^48^, we are not aware of any published works that use this technique to investigate the nutritional ecology of other brown bear populations in Europe.

In order to apply SIA, invasively collected tissue samples are costly, provide limited sample sizes, and are impractical when dealing with endangered populations^49^. Alternatively, non-invasive sampling is ideal for SIA applications^26,27,42,50^, especially for species whose hairs, vibrissae or feathers are relatively easy to collect^26,27,51,52^, and bears are no exception^53–55^. In particular, hairs act as an archive of past assimilated foods and can be harvested by non-invasive sampling or conveniently obtained through DNA-based population surveys^55,56^, where isotopic profiles can be reconstructed for individual bears^18,20^. Hairs are metabolically inert and the neosynthesized hair portion reflects the more recently assimilated diet^57,58^, not being affected by successive variation that may occur in the diet^26,59,60^. Therefore, a temporal sequence in diet composition can be retrospectively read across hair sections, progressing from the tip to the root of the hair^57^. Bears have a single annual moult, occurring in a lapse of time that spans from spring to summer^40,60–62^, and hair growth, at an approximate rate of 15 mm/month, lasts for the entire period of activity each year, from the moult until dormancy^40,60–62^. Therefore, comparing isotopic values of sequential sections of the hair provide a means to investigate variation in the diet that may take place during the hair growth period^26,57,59,60,63^. Accordingly, stable isotope analysis of hair sections has been successfully used to reconstruct bear diets^40,64,65^, detect temporal shifts in the diet^21,25,44,66,67^, and identify individual bears habituated to anthropogenic foods^20,21,26,27,42^.

**Figure 1:**
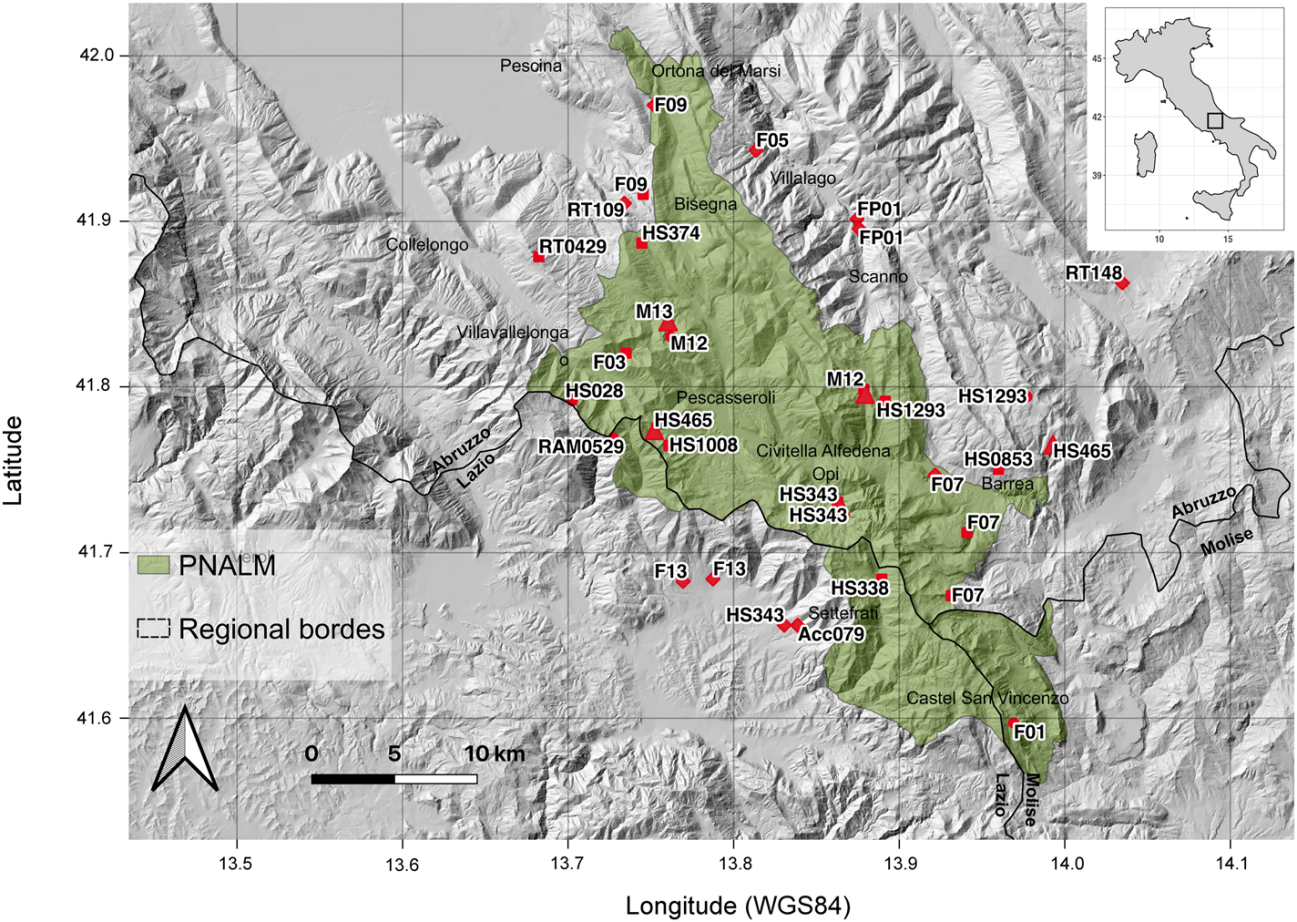
Distribution of 35 bear hair samples used to apply Stable Isotope Analysis to assess the assimilated diet of Apennine brown bears. Hair samples were collected through four sampling strategies (Acc: accidental sampling, HS: hair-snagging; RAM: hair snagging at *Rhamnus* patches; RT: hair snagging at rub trees.) during a survey of the bear population in the Abruzzo Lazio and Molise National Park (Central Italy, June–September 2014).

Apennine brown bears (*Ursus arctos marsicanus*) survive as a relict and isolated autochthonous population of central Italy (Fig. 1), and are considered critically endangered at the European^68^ and the national^69^ scales. Despite long-time protection and conservation efforts, Apennine brown bears have not showed clear signs of recovery and range expansion in the past decades^70,71^, despite the availability of suitable habitat at its connectivity at the landscape scale^72^. In addition to elevated risks of human-caused mortality, accumulated deleterious mutations^73^ and a persistently small population size contribute to a substantial risk of extinction in the medium term, especially if environmental stochasticity is taken into account^74^. Similarly to other small and isolated bear populations^75,76^, a fundamental conservation strategy rests on habitat management to support range expansion and to ensure long term productivity of the habitat.

Apennine bears feed on a large variety of herbaceous plants, wild fleshy fruits and hard mast, and show an important dependency on beechnuts^13^. Ants also represent a high-energy rich key-food especially during the summer^77^, while wild mammals (mainly ungulates) are consumed in the spring, though in lower proportions compared to other European brown bear populations. Thus, habitat productivity, heterogeneity of food sources, the close coexistence with humans and a variable interindividual diet can be critical for the survival of the Apennine brown bear population. In particular, previous investigations of the food habits of Apennine bears left unanswered relevant questions, namely^13^: (i) the extent of gender and individual variation in the diet^23^; (ii) the potential underestimation of meat consumption^15,17,78^; and (iii) the relative contribution of wild vs cultivated fleshy fruits in the diet, especially during fall. In addition, as depredation on livestock, crops and beehives occasionally occur in this bear population, where food-conditioned bears are being increasingly reported (P. Ciucci, pers. comm.), we were also interested in assessing the practical adoption of SIA to determine the dependency by individual bears on anthropogenic sources^20,21,45^.

We applied SIA to the Apennine bear population by taking advantage of individually DNA-identified hair samples collected in a 2014 non-invasive survey^54^. This provided the opportunity to extend and complement our current knowledge on the nutritional ecology of Apennine bears by testing the following research predictions: (i) the assimilated diet of adult Apennine female bears, according to their smaller size, differs from that of adult male bears^23,39^; (ii) the contribution of large mammals (i.e., wild and domestic ungulates) to the assimilated diet is higher, especially during spring, with respect to what previously determined from scat analysis^13,39^; (iii) due to the richness and diversity of fruit-bearing plants in the study area, the relative contribution of cultivated vs wild fleshy fruits to the assimilated diet is negligible; (iv) similar to SIA investigations in other bear populations with food-conditioned bears^20,21,45^, stable isotope profiles of Apennine bears allow identification of individuals with a higher degree of dependency on anthropogenic food sources. By applying SIA through hair section analysis, and accounting for both individual variation and natural vs anthropogenic foods, we exemplify how this technique con improve inference about diets of mammal species in a wide variety of systems.

## Results

### Isotopic signatures of bear key-foods and hair samples

**Figure 2:**
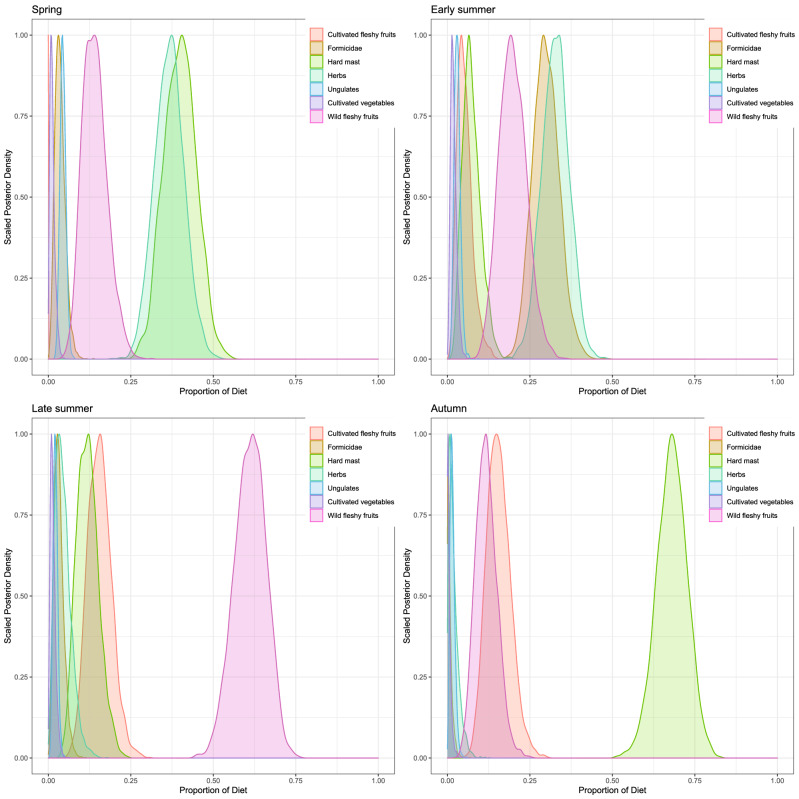
Distributions of posterior probabilities of each diet type for Apennine brow bears provided by stable isotope mixing models with informative priors (Abruzzo Lazio and Molise National Park, central Italy, 2013–2014).

We observed differences in the isotopic composition of bear key foods for both δ^13^C (ANOVA, Factor = “Food Category”, F_7,189_ = 76.51, p < 0.001, explained deviance ≈ 73%) and δ^15^N (ANOVA, Factor = “Food Category”, F_7,189_ = 64.56, p < 0.001, explained deviance ≈ 69%). In particular, herbs had the lowest δ^13^C and δ^15^N values, whereas cultivated vegetables had the highest δ^15^N values (Table 1; Fig. 2). Values of δ^13^C wild fleshy fruits, cultivated fleshy fruits, and hard masts were not different from each other (Tukey’s pairwise comparisons, p-value > 0.05 for both post-hoc comparisons). However, δ^15^N values of cultivated fleshy fruits were higher than both wild fleshy fruits (p-value < 0.05) and hard mast (p-value < 0.05). Ungulates had higher δ^13^C and δ^15^N values than wild fleshy fruits, cultivated fleshy fruit, and hard masts (p-values always < 0.01, except δ^13^C of hard masts p-value < 0.05). Furthermore, they had similar δ^13^C (p-value > 0.05) but higher δ^15^N compared to ants (p-value < 0.05).

**Table 1:** Mean isotopic ratios and elemental concentrations for sampled Apennine bear key foods in the National Park of Abruzzo Lazio and Molise, central Italy.

	δ^13^C (‰)		δ^15^N (‰)		[C]		[N]		Sample
	Mean	SD	Mean	SD	Mean	SD	Mean	SD	size
**Herbs**	**− 30.1**	**1.6**	**− 2.2**	**1.8**	**45.9**	**1.7**	**2.7**	**1.1**	**63**
*Chenopodium bonus-henricus*	− 28.8	0.8	− 2.3	1.4	46.2	1.0	5.1	1.4	3
Compositae	− 31.0	1.9	− 2.2	1.8	45.2	1.8	2.4	0.6	13
Graminacea	− 29.7	1.4	− 2.7	1.8	46.2	1.9	2.4	1.3	28
*Trifolium thalii*	− 30.3	1.1	− 1.0	0.6	45.7	1.4	3.4	0.6	9
Umbrelliferae	− 30.2	1.9	− 1.9	2.4	45.5	1.3	2.3	0.3	10
**Hard masts**	**− 27.6**	**1.5**	**− 0.7**	**2.1**	**50.4**	**7.7**	**2.1**	**1.4**	**18**
*Fagus sylvatica*	− 28.1	1.9	− 0.8	2.0	54.6	4.0	3.7	0.8	6
*Quercus cerris*	− 27.2	2.0	− 3.2	0.9	45.6	0.6	0.7	0.1	6
*Quercus pubescens*	− 27.6	0.8	0.4	1.7	44.9	1.0	1.4	1.2	6
**Wild fleshy fruits**	**− 27.8**	**1.5**	**− 2.6**	**2.1**	**46.8**	**2.9**	**0.8**	**0.4**	**46**
*Cornus mas*	− 28.2	2.1	− 5.6	0.6	42.8	0.3	0.4	0.1	3
*Crataegus monogyna*	− 26.7	0.8	− 3.0	1.0	44.4	0.9	0.6	0.1	4
*Fragaria vesca*	− 29.1	2.4	− 3.0	2.7	48.8	1.5	1.4	0.3	5
*Malus sylvestris*	− 26.9	0.9	− 3.2	1.3	44.9	0.6	0.4	0.2	5
*Prunus spinosa*	− 28.2	1.0	− 1.9	2.5	48.9	1.8	0.7	0.4	4
*Pyrus pyraster*	− 27.3	0.1	− 1.9	1.3	46.7	3.0	0.6	0.0	3
*Rhamnus alpina*	− 27.4	1.0	− 2.4	2.0	45.2	0.7	0.7	0.1	7
*Rosa canina*	− 26.2	0.8	− 2.3	3.5	45.0	1.2	0.9	0.3	5
*Rubus idaeus*	− 29.9	1.2	− 3.6	1.6	51.0	1.3	1.3	0.2	4
*Rubus ulmifolius*	− 28.6	0.9	− 0.9	1.7	49.4	2.8	1.2	0.3	6
**Cultivated fleshy fruits**	**− 27.2**	**1.4**	**0.6**	**2.4**	**43.5**	**1.2**	**0.6**	**0.4**	**16**
*Ficus carica*	− 28.5	0.3	− 1.5	0.2	45.4	1.3	1.0	0.2	3
*Malus domestica*	− 25.8	1.5	0.5	2.3	43.5	0.3	0.4	0.2	4
*Prunus domestica*	− 28.3	1.5	3.7	1.3	43.0	0.5	0.9	0.4	3
*Pyrus communis*	− 26.9	0.6	0.1	2.3	42.7	0.7	0.3	0.1	6
**Cultivated vegetables**	**− 28.5**	**1.5**	**9.0**	**3.3**	**38.0**	**3.7**	**294**	**1.3**	**12**
*Cichorium intybus*	− 28.5	0.3	10.6	3.2	37.2	2.3	2.5	0.4	3
*Daucus carota*	− 26.4	1.0	4.4	0.5	40.7	1.5	1.4	0.2	3
*Eruca vesicaria*	− 29.0	0.3	10.5	0.7	34.7	4.0	4.2	0.7	3
*Lactuga sativa*	− 30.0	0.1	10.5	2.7	39.3	4.4	3.5	1.6	3
**Ungulates**	**− 25.1**	**1.3**	**4.3**	**2.0**	**49.9**	**4.5**	**4.0**	**2.1**	**7**
*Bos taurus*	− 25.5	0.2	2.2	0.6	49.3	1.1	3.6	0.2	1
*Capra hircus*	− 24.1	0.0	3.7	0.2	48.1	0.7	3.5	0.0	1
*Cervus elaphus*	− 26.3	1.0	3.9	1.4	53.2	6.6	5.2	3.5	3
*Ovis aries*	− 23.1	0.0	7.8	0.9	46.2	0.9	3.4	0.1	1
*Sus scrofa*	− 25.3	0.0	4.5	0.3	49.1	1.5	3.5	0.1	1
**Formicidae**	**− 26.6**	**1.2**	**1.7**	**1.1**	**52.0**	**5.5**	**9.4**	**1.7**	**26**
**C4—*Zea mays***	**− 11.9**	**0.4**	**4.3**	**1.5**	**45.6**	**0.5**	**17.4**	**2.3**	**3**

### Isotopic signatures of bear hair sections and seasonal diet

**Figure 3:**
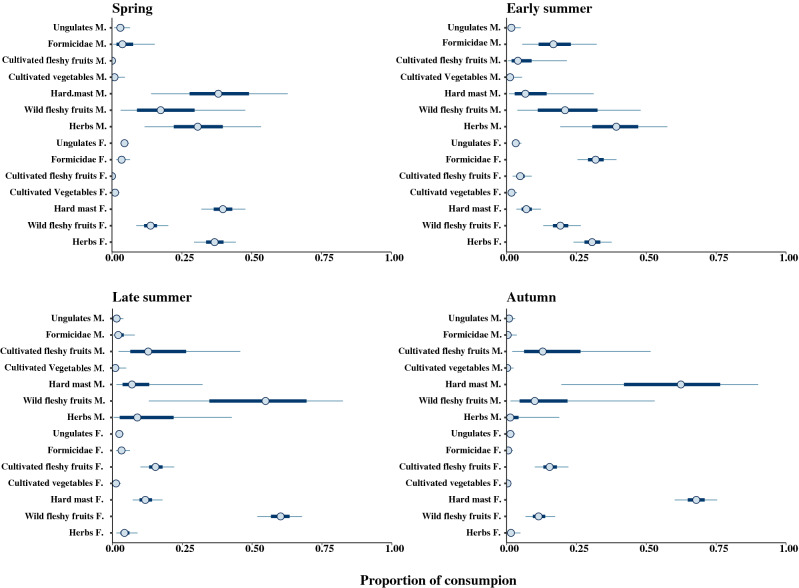
Mean estimated posterior proportional dietary contribution for male (M) and female (F) Apennine Brown bears in the four dietary seasons (Abruzzo Lazio and Molise National Park, central Italy, June–September 2014). Circles represent the median contribution, of each key-food category, to bears diet, with 95% (thin lines) and 50% (thick lines) credibility intervals.

Overall mean stable isotopic values of hair sections per individual bear were δ^13^C = − 22.1 ± 0.7‰ and δ^15^N = 3.1 ± 1.7‰, and we found no differences in the mean δ^13^C and δ^15^N values between hair sections sampled pre- and post-moult (ANOVA, Factors = “Sampling period”, F_1,193_ = 0.03, p > 0.5). We detected no differences in δ ^13^C values across seasons and sexes, although we revealed differences among individual bears (ANOVA, Factors = “BearID”, F_26,174_ = 7.9, p < 0.001, explained deviance ≈ 54%). However, we revealed differences in δ ^15^N values across seasons, sexes and individual bears (ANOVA, Factors = “Season + Sex + BearID”, F_29,165_ = 10.72, p < 0.001, explained deviance ≈ 60%). In spring and autumn, δ ^15^N values were lower compared with other seasons, and δ ^15^N values of male bears in these two seasons were higher compared with female bears (p < 0.005) (Table 2). Due to the particularly high δ^13^C values of C4 plants (Table 1), we failed to reveal corn consumption by the bears we sampled (Fig. 2). All the isotopic signature of the hair sections, except 2 (successively removed from the models), fell within the 95% mixing region (Supplementary Figure 1). In all seasons, the models that included the covariate “BearID” were the most supported (Table 3). At the population level, the estimated mean probability of consumption of herbs was 34.1% (CI 25.7–43.3%) in spring and 24.6% (CI 16.2–34.2%) in early summer, but was null in late summer and autumn (Table 4). According to the model “Sex + BearID” (Table 3), in spring, herbs appeared to be consumed more abundantly by female bears (33.6%; CI 24.7–43.1%) than males (25.9%; CI 3.8–65.3%) (Fig. 3). The probability of bears consuming cultivated vegetables was null in all seasons (Table 4).

**Table 2:** Mean carbon and nitrogen isotopic values across hair sections of Apennine brown bears according to gender and dietary season (Abruzzo Lazio and Molise National Park, central Italy, 2013–2014).

Season	Sex	δ^13^C (‰)		δ^15^N (‰)		No of bears	No of hair sections
		Mean	SD	Mean	SD		
Spring	F	− 22.0	0.8	2.9	1.2	14	45
	M	− 22.0	0.7	3.5	0.8	5	9
Early summer	F	− 22.1	0.7	3.6	1.1	13	40
	M	− 22.4	0.6	3.8	0.9	11	21
Late summer	F	− 22.1	0.8	3.2	1.8	16	33
	M	− 22.6	0.6	3.5	1.9	8	22
Autumn	F	− 22.0	0.6	1.7	2.3	6	14
	M	− 22.4	0.7	2.7	2.4	6	12

**Table 3:** Model selection of seasonal sets of Stable Isotope Mixing Models to assess factors affecting the assimilated diet of Apennine bears (June–September 2014, Abruzzo Lazio and Molise National Park, central Italy).

Season	Model structure	LOO_ic_	SE LOO_ic_	ΔLOO_ic_	SE ΔLOO_ic_	Weight
**Spring**	Status + BearID	168	23.5	0	–	**0.548**
	Sex + BearID	169.1	22.2	1.1	3.8	**0.316**
	BearID	170.8	23.3	2.8	2.8	0.135
	Sex + Status	197.6	29.3	29.6	12.1	0
	Status	198.3	28.5	30.3	11.4	0
	Null	207.1	25.4	39.1	12.1	0
	Sex	210.3	24.9	42.3	11.8	0
**Early summer**	Status + BearID	176.8	16.9	0	–	**0.698**
	BearID	179.1	17.4	2.3	3.5	**0.221**
	Sex + BearID	181.1	17.3	4.3	3.7	0.081
	Sex + Status	221.2	19.6	44.4	10.6	0
	Status	226.9	21.3	50.1	10.7	0
	Sex	238.6	23.8	61.8	13	0
	Null	241.9	26.1	65.1	15.2	0
**Late summer**	BearID	210.7	16.7	0	–	**0.558**
	Sex + BearID	211.4	16.2	0.7	1	**0.393**
	Status + BearID	215.6	16.1	4.9	5.2	0.048
	Sex + Status	223.3	17.1	12.6	9.7	0.001
	Status	237.8	17.7	27.1	10.6	0
	Null	255.5	18.2	44.8	6.1	0
	Sex	257.8	17.9	47.1	6.3	0
**Autumn**	Status + BearID	81.8	9	0	–	**0.362**
	Sex + BearID	82	9	0.2	0.5	**0.327**
	BearID	82.1	9.2	0.3	0.6	**0.311**
	Sex + Status	120	9.4	38.2	4.7	0
	Status	122.7	9.4	40.9	5.1	0
	Null	127.2	9.3	45.4	3.7	0
	Sex	128.4	9.2	46.6	3.3	0

LOO_ic_: LOO information criterion; SE LOO_ic_: standard error of LOO_ic_; **Δ**LOO_ic_: difference between each model and the model with lowest LOO_ic_; SE **Δ**LOO_ic_: standard error of the difference between each model and the model with lowest LOO_ic_; Weight: relative support for each model, calculated as Akaike weights. Akaike weights > 0.2 were highlighted in bold.

**Table 4:** Mixing model estimated dietary proportions of key foods categories for Apennine brown bears (Abruzzo Lazio and Molise National Park, central Italy, 2013–2014).

Key food category	Spring			Early summer			Late summer			Autumn		
	Mean %	2.5%	97.5%	Mean %	2.5%	97.5%	Mean %	2.5%	97.5%	Mean %	2.5%	97.5%
Hard mast	39.1	29.9	48.3	7.9	3.3	14.5	11.7	6.2	18.7	67.4	57.6	76.1
Cultivated fleshy fruits	0	0	0	5.1	1.7	10.2	15.2	9	22.8	15.6	9.1	23.3
Wild fleshy fruits	13.7	7.7	21.1	21.0	13.2	30	59.2	49.3	68.4	10.9	5.6	17.5
Ungulates	7.4	3.8	12.9	4.6	2	9.5	3.3	1.3	6.4	2.8	0.7	6.8
Herbs	34.1	25.7	43.3	24.6	16.2	34.2	5.0	1.3	10.1	1.8	0.2	5.4
Formicidae	4.4	1.4	8.8	34.6	25.8	43.9	4.2	1.3	8.5	0.9	0	3.2
Cultivated vegetables	1.3	0.2	3.4	2.1	0.5	4.9	1.4	0.3	3.6	0.6	0	2.5

Values are reported as mean probabilities and upper and lower 95% credibility intervals. Food categories are ordered in descending order relative to Autumn.

The mean probability of bears consuming ungulates was overall relatively low, with highest values observed in spring (7.4%; CI 3.8–12.9%) followed by early summer (4.6%; CI 2.0–9.5%), but null in late summer and autumn (Table 4), consistently between sexes, and individual bears.

Estimated ant consumption was marked in early summer (36.6%; 25.8–43.9%) but negligible in the other seasons. According to the models “Sex + BearID” (Table 3), during summer consumption of ants tended to be higher for female (34.5%; CI 25.9–43.5%) than male (19.1%; 4.2–39.9%) bears (Fig. 3).

Wild fleshy fruits were consumed mostly in late summer (59.2%; CI 49.3–68.4%) and in lower amount in early summer (21.0%; CI 13.2–30.0%) and autumn (10.9%; CI 5.6.1–17.5%) (Table 4), with no differences between female and males (Fig. 3). Cultivated fleshy fruits were consumed only in late summer (15.2%; CI 9.0–22.8%) and autumn (15.6%; CI 9.1–23.3%) (Table 4), without differences between sexes (Fig. 3). The estimated consumption of hard mast for the population peaked in autumn (67.4%; CI 57.6–76.1%), followed by spring (39.1%; CI 29.9–48.3%) and late summer (11.7%; CI 6.2–18.7%) (Table 4). During fall, males tended to exploit less hard mast (57.6%; CI 13.7–92.8%) than females (67.5%; CI 58.1–76.3%), while these differences waned in spring (Fig. 3).

Mean δ^13^C values of individual bears ranged from − 23.5 ± 0.3‰ to − 21 ± 0.3‰ (Supplementary Table S1), while mean δ^15^N values ranged from − 0.5 ± 0.6‰ to 5.4 ± 0.5‰ (Supplementary Table S1). We revealed remarkable individual variation in the consumption of herbs in spring, that ranged from 15.5% (CI 0.7–51.9%), for one food-conditioned female (FP01), to 73.3% (CI 5.7–95.1%; Supplementary Table S2). In late summer, the probability of wild fleshy fruits consumption was lowest for bear FP01 (33.9%, CI 2.1–77.4%) but highest for three non-management bears (F03: 81.9%, CI 16.4–98.7%; HS0853: 86.3%, CI 14.5–99.1%; M12: 87.7%, CI 10.9–99.2%) (Supplementary Table S2). In autumn, the proportion of cultivated fleshy fruits ranged from 2.4% (CI 0–14.5%) for bear M12 to 54.7% (CI 0–100%) for bear HS465 (Supplementary Table S2). Concerning hard mast, consumption in autumn ranged from 2.9% (CI 0–20.7%) for bear HS465 to 83.5% (CI 0–100%) for bear M12 (Supplementary Table S2), and in spring from 12.3% (CI 0.4–77.2%) for bear F09 to 60.3% (CI 8–50.4%) for bear F01(Supplementary Table S2).

**Figure 4:**
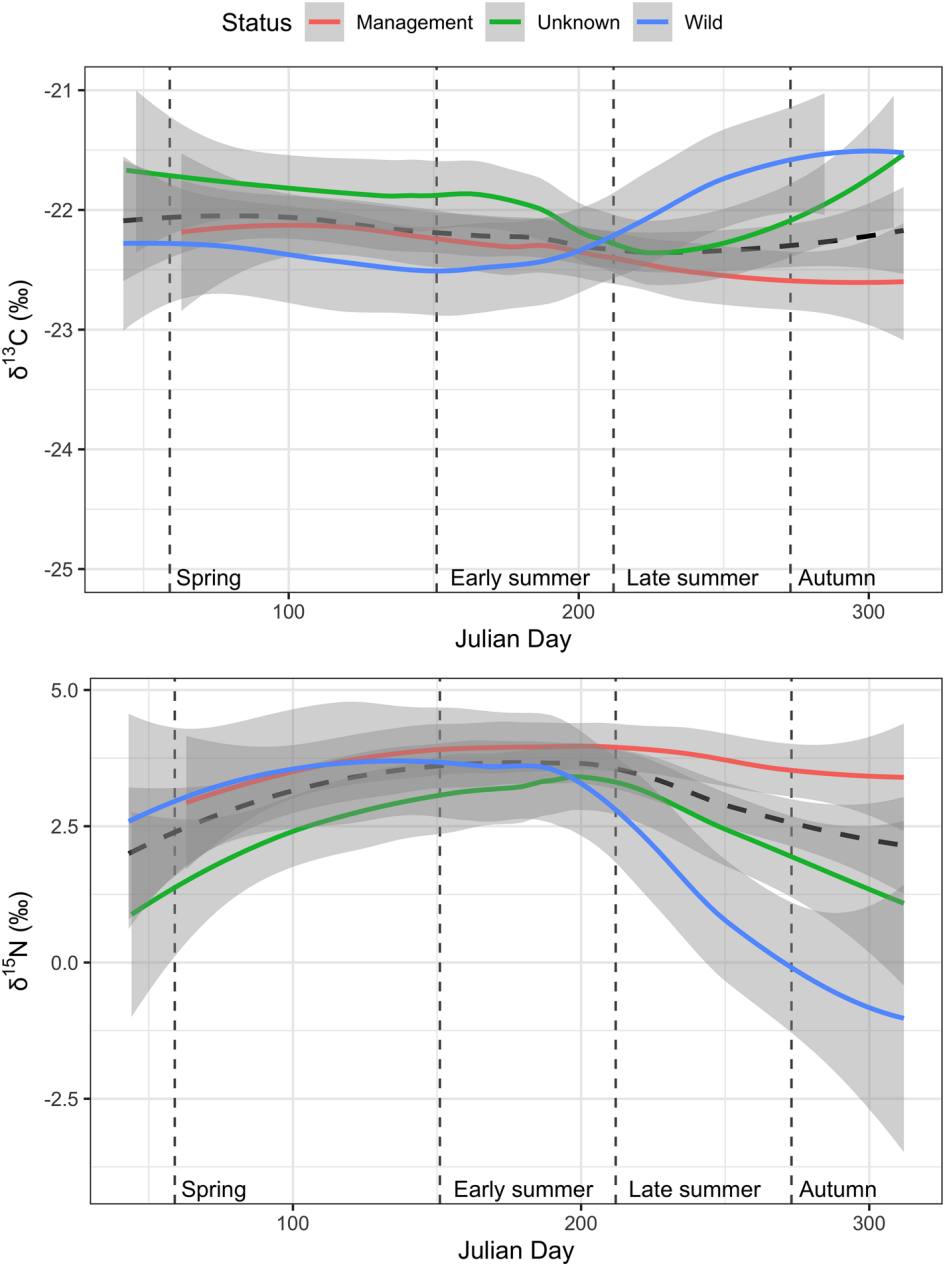
Stable isotope values (δ^13^C, upper panel, and δ^15^N, lower panel) of Apennine bear hair sections across the activity period (Abruzzo Lazio and Molise National Park, central Italy, June–September 2014). Dashed line indicates smoothed mean for the population and the grey shaded area its 95% confidence interval.

**Figure 5:**
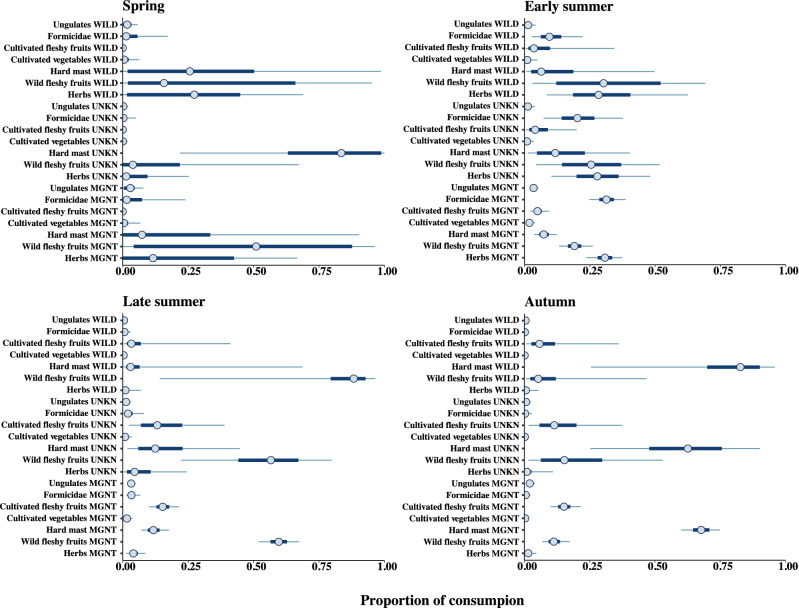
Mean estimated posterior proportional dietary contribution for wild (WILD), unknown (UNKN) and management (MGNT) Apennine Brown bears in four dietary seasons (Abruzzo Lazio and Molise National Park, central Italy, June–September 2014). Circles represent the median contribution, of each key-food category, to bears diet, with 95% (thin lines) and 50% (thick lines) credibility intervals.

We observed differences in δ ^13^C and δ ^15^N isotopic signatures between management and the other bears, with the former being 0.9‰ lower in δ ^13^C (ANOVA, Factors = “Season * Status”, F_7,187_ = 2.67, p < 0.05, explained deviance ≈ 5%) but 2.9‰ higher in δ ^15^N compared to non-management bears, in particular during fall (Fig. 4; ANOVA, Factors = “Season * Status”, F_7,187_ = 10.01, p < 0.001, explained deviance ≈ 25%). According to the model “Status”, compared to than management bears non-management bears exploited more herbs in spring (27.6%, CI 0–75.0%), more wild fleshy fruits both in early (33.0%, CI 2.1–74.4%) and late (78.5%, CI 7.7–97.4%) summer, and more hard mast in autumn (76.2%, 20.5–96.9%) (Fig. 5). On the other hand, management bears tended to consume greater proportions of meat (3.6%, CI 1.8–6.0%) in early summer, and of cultivated fleshy fruits both in late summer (15.5%, CI 9.0–22.8%) and autumn (15.4%, CI 9.3–23.1%) (Fig. 5).

## Discussion

This is the first application of stable isotopes analysis to the study of the feeding habits of the Apennine brown bears, and our findings functionally complement previous dietary knowledge based on traditional scat analysis^13^. Applying SIA to guard hair sections of Apennine bears allowed us to reconstruct their assimilated diet with an enhanced seasonal and individual resolution. Our findings confirm the importance of key foods, such as hard mast during autumn, fleshy fruits in late summer, and ants during early summer, for the maintenance and growth of bear tissues. By using hair sections of previously genotyped individual bears, we have found indications of dietary differences between female and male bears, confirmed a low contribution of meat (ungulates) compared to other brown bear populations, and failed to find a strong dependency on anthropogenic foods. Nevertheless, a single year of sampling and relatively small sample sizes for some season and sex/management status combinations, coupled with small effect sizes, somehow require cautious interpretation of some of our findings. By sampling in 2014 only, our diet description reflects conditions specifically met in the year of sampling and does not capture the expected year-to-year variation in foods available to bears. For example, the relatively high consumption of hard mast in spring that we reported (Table 4) is unlikely to occur in a typical year, except following mast years when overwintering hard mast may still be available to bears from the previous fall^13^. Likewise, the differences we found in the assimilated diet between male and female bears, or between bears of different management status, suffer from considerable uncertainty, as exemplified by the large overlap between the credibility intervals we reported (cf. Fig. 3). While these results hint at likely important differences, stronger statistical inference would benefit from longer, replicated studies and greater sample sizes.

SIA has been previously applied to several dietary studies on American black bears (*Ursus americanus*), Asiatic black bears, and brown bears^20,26–28,35,60^. Compared to these studies, and in particular to those comprising isotopically well separated items such as salmon^19,65,66,79^ or C4 plants^21,42^, the results of our mixing models were less obvious, although we sampled all key foods of Apennine brown bears. This is due to the similarity in the δ^13^C and δ^15^N values among the food sources that contribute to the Apennine bear’s diet, as also revealed in similar ecological conditions for brown bears in Slovenia^48^. Nevertheless, using adequate priors in mixing models we alleviated this issue, estimating the proportion of assimilated food sources with a seasonal resolution by individually-recognized bears. However, when dealing with stable isotope data, some degree of uncertainty is inherent in SIA, including natural variability in the sources’ isotopic signatures, variable TEF within a given species, different stable isotope ratios in different tissues, variations in the isotopic source and mixture process errors^80^. It has been recently recognized that the use of informative priors may introduce bias in the posterior estimations^81,82^, leading to erroneous representations of diet composition^83–85^. Nevertheless, we believe that incorporating informative priors based on non-isotopic data, such as prior knowledge on diet composition obtained with traditional dietary methods, in some cases can improve the precision of dietary reconstruction by SIMMs. This is especially true when dealing with food sources that overlap largely in the isotopic mixing space, as in the case we observed with hard mast, wild fleshy fruits, and cultivated fleshy fruits. In addition, when multiple sources have similar isotopic values, uninformative priors may equate each of these sources, rendering them of similar importance in the diet and resulting in a large degree of uncertainty^86^. Accordingly, we observed similar effects with herbs, hard mast, wild fleshy fruits, and cultivated fleshy fruits in our study area, all resources whose estimated proportion in the diet would have been the same had we used uninformative priors (Supplementary Figure S2). In addition, the adoption of uninformative priors can overestimate the consumption of a resource also in seasons when it is not available, as it might be the case with resources that are available for short bursts of time (i.e., ants in the bear summer diet^13,77,87^). We therefore believe that, in our case, models with informative priors are better suited to provide a more realistic estimation of the bear diet, and this is in line with evidence from other studies^88^, including those based on experimental feeding trials^89,90^. Moreover, in our case the adoption of informative priors allowed us to draw inference about the likely difference between the assimilated diet of male and female bears, or between that of management and non-management bears (covariates not directly influenced by priors), which was a relevant research question of our work.

Although we obtained indications of likely dietary differences between sexes and between management and non-management bears, most bears showed high variance in the δ^13^C and δ^15^N values and significant inter-individual differences in their seasonal isotopic patterns. Vegetable matter (herbs, fleshy fruits and hard mast) represented the major component of the assimilated diet across the dietary seasons, but neither C4 plants (corn) nor vegetable crops emerged as staple and recurrent foods for Apennine bears.

Brown bears are opportunistic consumers, and their trophic preferences are strictly related to the seasonal presence and abundance of food resources across multiple trophic levels^7^. Apennine bears are no exception and feed primarily on plant matter and acorns^13^. We did not observe any important contribution of terrestrial meat sources, such as wild ungulates or livestock, nor of vegetables derived from crops and gardens. Although we failed to detect differences in the consumption of ungulates by male and female bears, the high nitrogen isotopic signatures in male bears during spring and autumn could indeed be related to a major intake of animal protein by male bears. As expected, the consumption of ungulates by Apennine bears, and in particular by dominant male bears, can reflect scavenging on winter-killed ungulates or abandoned livestock carcasses^91^. Bear predation on free-ranging sheep and calves do occur in the Abruzzo Lazio and Molise National Park (PNALM) mostly during summer^13,92^, but in our analysis we could not discriminate between wild and domestic ungulates due to the similarity in their isotopic signatures. Compared to other European bears^93,94^, the low consumption of ungulates by Apennine bears can account for their smaller size, possibly enabling them to meet their energy requirements feeding at lower trophic levels than larger bears^95–97^. Moreover, we did not report any evidence of consumption of vegetable from farming. Isotopic signal of cultivated vegetables analyzed in this study were very high in δ^15^N, compared both to natural plant resources and to isotopic signal of bear hair sections, possibly accounting for the use of organic fertilizers^98,99^. Since none of the bears we sampled reflected such high δ^15^N values, it is reasonable to expect that cultivated vegetables are not largely consumed in this bear population. Furthermore, in early summer, our findings suggest a higher consumption of ants by females compared to males. The use of ants as food by bears occurs essentially during this season, when the availability of ants and their brood increased^77,100–102^. Our observations are congruent with a previous scat-analysis study according to which ants were estimated to provide an average of 35.7% of digestible energy to Apennine brown bears^77^, and with reports for brown and black bears^8,100,103–106^. Ants are rich in protein (up to 50%) and thus are an important source of essential amino acids^100,107,108^. They represent a consistent and easy-accessible food source, contributing to meet the additional energy and protein requirements for lactation and cub growth^108^. A greater ant consumption by female bears, as our findings seem to indicate, may also reduce intraspecific competition with males, thus mitigating the risk of infanticide^18,19,22,39^. Fleshy fruits were another important key food for Apennine bears, especially in late summer, when hard mast is not yet completely ripe, or also in autumn in mast-failure years^13^. Although fleshy fruits can be an excellent source of energy due to their high carbohydrate content, they can be very low in protein, in specific amino acids, or in other nutrients^109^. Fruits usually contain between 3 and 7% of crude protein, which is below the minimum/optimum dietary protein requirement (≈ 12–17%)^109,110^. This could explain why bears are inclined to consume fruits as part of a mixed diet, suggesting an optimization process between the cost of maintenance and the energy requirement, in accordance with the optimal foraging theory^111^.

Using SIA we were also able to discriminate between consumption of wild vs cultivated fleshy fruits, a crucial point not clarified in previous dietary studies^13^. Cultivated fleshy fruits had higher δ^15^N and δ^13^C values than wild fruits, likely revealing the use of organic fertilizers similarly to cultivated vegetables^98,99^. Based on this, we found that, although with high inter-individual variation, Apennine bears consumed up to 15% of cultivated fleshy fruits both in late summer and autumn, representing 20.3–66%, respectively, of all fleshy fruits consumed in these seasons. Despite the PNALM is a productive ecosystem of wild fruits for bears, such as *Rhamnus,* wild rose, wild pears and other wild fruit ripening during the hyperphagic phase^13^, the consumption of cultivated fleshy fruits that we revealed could be due to the availability of abandoned or easily accessible cultivations^70^.

Another relevant insight offered by our SIA application is the likely difference in δ^15^N and δ^13^C values between management and non-management bears. While the two bear categories shared similar isotopic values in spring, their difference was increasingly marked for the rest of the active period. During spring, herbaceous vegetation is an important source of energy and protein, and in particular newly sprouted plants that are rich in nutrients and protein and lower in fiber^112–115^. In this season, both management and non-management bears are offered a similar diet of newly grown herbaceous vegetation and a few overwintered carcasses of ungulates, with few anthropogenic foods still available compared to other seasons. In late summer and autumn, however, while non-management bears preferentially foraged on fleshy fruits and hard mast, management bears appeared to use a richer array of anthropogenic food sources as they became increasingly available. Despite no direct measures of habitat productivity are available in the PNALM, no evidence supports the hypothesis that food limits the bear population in the study area^70^. However, limited bear depredation of crops, livestock, poultry and domestic rabbits has been an issue since the late 1960s^70,92^. As observed in other studies^20,26,27,42^, our findings indicate that management bears that fed on anthropogenic food sources tended to have higher δ^15^N than non-management bears. For example, one management bear (FP01) had the highest δ^15^N value during the entire year, and this female is well known to repeatedly visit villages for anthropogenic foods, in particular poultry (P. Ciucci, pers. comm.), whose isotopic signature is confounded in our analysis with those of ants and fleshy fruits. Similarly, another management bear (HS456), sampled by park wardens at a depredation event on poultry and beehives, also showed high δ^15^N values, and this bear’s genotype is compatible with one of FP01’s cubs. On the contrary, some non-management bears (e.g., M12 or F03) had the lowest δ^15^N values, indicating a consumption of natural resources as confirmed by our mixing models. Nevertheless, being compounded by a large individual variability, a small effect size, and a relatively large statistical uncertainty, the difference in isotopic signatures does not currently allow a reliable classification of the management status of individual bears, warranting further research based on larger and more focused sampling.

Maintaining the long-term diversity and accessibility of foods to bears is a primary management goal for the conservation of this endangered population. According to our and previous findings, habitat management should aim to maintain abundance of mature stands of hard mast producing species, while ensuring the sustained availability of other seasonal key foods^13^. We suggest that productivity of ants should be also carefully considered within forest and land habitat management practices, as they represent a key food especially for female bears, therefore potentially impacting fertility and population productivity. We also stress the importance of facilitating accessibility by bears in spring and early summer to lowland drainages that contain abundant grasses and forbs^13^.

Further studies should be conducted to better understand some unsolved aspects of the feeding ecology of Apennine bears, and in particular the relative contribution of wild vs domestic ungulates or the contribution of other anthropogenic foods (i.e., poultry, beehives, crops). In addition, future SIA applications to this bear population could benefit from enhanced statistical power by adding to mixing models covariates such as age and body condition, by using additional tracers (e.g., δ^34^S^25,40,50^), by including a larger number of management bears in the sample, and by adopting longer study periods to account for inter-annual variability in the productivity of bear key foods.

## Methods

### Ethics statement

Protocols to collect bear samples and handling procedures were agreed with the PNALM authority in accordance with international guidelines^116^, following official permits by the Italian Ministry of the Environment. Similarly, plants and vegetal material were collected in accordance with the PNALM authority.

### Study area

Our study area corresponds to the core distribution of the Apennine brown bear population, including the National Park of Abruzzo Lazio and Molise (PNALM) and adjacent areas, located in the central Apennines, Italy^71^ (Fig. 1). Elevation ranges from 400 to 2285 m and the terrain is typically mountainous with Mediterranean mountain climate^117^. The area is mostly covered by deciduous forests (about 60%), followed by subalpine meadows and grasslands, with crops nearby villages and mostly along valley bottom. Large ungulates in the area comprise roe (*Capreolus capreolus*) and red deer (*Cervus elaphus*), wild boar (*Sus scrofa*), and Apennine chamois (*Rupicapra pyrenaica ornata*). Livestock is also grazed at relatively high densities especially during summer months. In addition to bears, wolves (*Canis lupus*) occur in the area^118^, and free-ranging dogs are occasionally present. In the years of the study, the brown bear population was estimated at about 50 (95% CI 45–69) bears, including cubs^54^, and seemed to have remained stable during the past decade^119^. Bears in the study area are generally active from mid-March to the end of November, with differences in denning chronology depending on gender and reproductive status of the females (modal dates: adult female bears 27 November; adult male bears 12 December; P. Ciucci, unpublished data).

### Hair and key foods sampling

Bear hairs were collected during a non-invasive population survey conducted in 2014^54^. To this aim, a sampling grid (cells of 5 × 5 km) was overlaid to the study area and hair collection (May–October 2014) occurred through systematic hair-snagging^53,55^, complemented by additional sampling methods (i.e., rub-tree sampling, opportunistic sampling at buckthorn patches, and incidental sampling^54^; Fig. 1). We defined a hair sample as a tuft of hairs with bulbs entangled in one set of barbs^53^, which we assumed belonged to the same bear, and collected each sample with gloves and sterilized surgical forceps to avoid contamination. Each sample was stored in a paper envelope labelled with a uniquely numbered barcode and then placed in a box with silica gel to prevent DNA degradation. From each sample, some hairs were then used for genetics analyses by clipping a few millimetres of the hair root including the bulb^54^, whereas intact hairs were used for stable isotope analyses. For the scope of the analysis, individual bears were further discriminated management bears, including a few bears known to be food conditioned or partly habituated to humans, from all other bears (i.e., non-management bears). To obtain isotopic values of foods available to bears, we sampled key foods consumed by Apennine bears at known bear foraging locations^13^, including (Table 1): (i) herbs, comprising graminoids, forbs and sedges; (ii) wild fleshy fruits; (iii) cultivated fleshy fruit (i.e., peer, apples, prunes); (iv) beechnuts (*Fagus sylvatica*) and acorns (*Quercus* spp.); (v) cultivated vegetables; (vi) ungulates, comprising wild and domestic ungulates; (vii) ants (various species), and (viii) C4 plants, comprising cultivated corn. All food samples were placed in plastic bags or tubes and stored at − 20 °C until treatment for SIA.

### Laboratory procedures and hair sectioning

A total of 35 bear hairs were processed for SIA, and they belonged to 27 different bears (16 females and 11 males). These included a few individuals known or suspected to be food-conditioned on the basis of ancillary information such as telemetry or non-invasive genetic sampling at damaged sites (Supplementary Table S1). We classified these bears as ’management bears’ (sensu Hopkins^120^), as opposed to ‘non-management bears’ or bears of ‘unknown’ status (Supplementary Table S1).

Hairs were washed with a sonic bath in a 2:1 chloroform–methanol solution to remove surface oils and impurities, and then dried at 60 °C in oven for at least 24 h^17,22^. Samples of bear key foods were freeze-dried and ground to a fine homogeneous powder using a ball mill (Fritsch Mini-Mill Pulverisette 23). Hairs were sectioned (see below), and all hair sections were singly weighed and closed into ultra-pure tin caps. Aliquots of sampled bear key foods were weighed twice and pressed into ultra-pure tin capsules^121,122^. All samples were analysed using a CN analyser (Vario Micro-Cube, Elementar Analysensysteme GmbH, Germany) coupled with an isotope ratio mass spectrometer (IsoPrime100, Isoprime Ltd., Cheadle Hulme, UK). Stable isotope ratios (^13^C:^12^C and ^15^N:^14^N) were expressed in delta notation (‰ deviation from international reference standards) in accordance with the equation:

$$\updelta {\text{R}}\left( \permil \right)\, = \,\left[ {\left( {{\text{R}}_{{{\text{SAMPLE}}}} {-}{\text{R}}_{{{\text{STANDARD}}}} } \right)/{\text{ R}}_{{{\text{STANDARD}}}} } \right]\, \times \,{\text{1}}0^{{\text{3}}} ,$$

where R is the heavy-to-light isotope ratio of the element^123^. The international standards were Vienna Pee Dee Belemnite (VPDB) for C and atmospheric N_2_ for N. The internal laboratory standard was IAEA-600 Caffeine. Measurement errors were found to be typically smaller than ± 0.05 for both δ^13^C and δ^15^N. Elemental concentrations were reported as % of the element on dry-weight.

To assign hairs (and their sections, see below) to their growth period, we assumed that fully grown hairs sampled before the moult (i.e., end of June) were grown during the previous activity period (i.e., previous year until dormancy), whereas hairs collected after the moult (i.e., end of August) were grown during the activity period of the current sampling year^20,21,40,43,60,61,64,65^. We included in the analysis only hairs collected in June (pre moult) and September (post moult), and excluded those sampled in July and August (i.e., during the moult) due to high uncertainty of their assignment. Starting from the root up to the tip of the hair, we cut guard-hairs with a surgical scalpel into 15-mm sections, which we assumed to correspond approximately to the hair growth of 1 month^40,61,62^. Specifically, for fully-grown hairs sampled in June we assigned the basal 15-mm section to the last month of growth before dormancy (November) in the previous year, the successive 15-mm section the month before (October), and so on. Instead, the basal 15-mm section of hairs sampled in September was assigned to the last month of growth before the actual sampling date, the successive 15-mm section to the month before and so on. However, to account for the inherent uncertainty in the monthly growth rate of hair sections^40,60–62^, we conducted the analysis at a seasonal resolution by pooling 15-mm hair sections across dietary seasons. These were defined based on the phenology of seasonal key foods for Apennine bears^13^: Spring, including sections whose growth was assigned to the months of March, April and May; Early summer, including sections assigned to June and July; Late Summer, including section assigned to August and September; Autumn, including hair sections assigned to October and November.

### Data analysis

To compare isotopic values of bear key foods, and to examine relationships between isotopic values and bear sex, season and year we used Analysis of Variance (ANOVA) followed by a post-hoc Tukey's HSD comparison test. All ANOVA models were tested for normality and homoschedasticy of residuals. Dietary proportions of assimilated key bear foods were estimated using Stable Isotope Mixing Models (SIMMs)^80^. Bear key food samples were a priori grouped into seven categories (Table 1) on the basis of their isotopic values and according to prior dietary knowledge from the same bear population^13^. We ran four separate seasonal models, with isotopic signatures of bear hairs as a mixture and isotopic signature of key-bear food categories as sources. In the above models we excluded C_4_ plants because this source did not match the mixing space^124^.

For each seasonal set of models, we contemplated different covariate structure, including the null model: a “Sex” model, with gender as a fixed effect; a “Status” model, with the management status as fixed effect; a “BearID” model, with individual bear ID as a random effect, and the combinations “Sex + Status”, “Sex + BearID” and “Status + BearID”. Since each set of models fit the same data, we performed model comparison using an information criterion approach, namely the leave-one-out cross-validation (LOO), which is more robust compared to the deviance information criterion (DIC), a generalization of the Akaike information criterion for Bayesian model selection^80,125^. In addition, we also used Akaike weights, using LOOic scores, which provide an estimate of the probability that one model will make the best predictions compared to the alternative models in the set^126^. We then selected models with the lowest LOO Information Criterion (LOO_ic_) and an Akaike weight > 20%^80,127^. Stable carbon and nitrogen isotope Trophic Enrichment Factors (TEFs) were obtained from the literature^24,28^. Because no study has accurately estimated the TEF of bear hairs, we used TEF values, expressed as Δ^13^C and Δ^15^N, estimated from a feeding experiment on Norway rats^28^. Therefore, for vegetable resources we added to δ^13^C and δ^15^N TEF values of Δ^13^C = 3.4 ± 0.5‰ and Δ^15^N = 2.4 ± 0.2‰, respectively, and for animal foods Δ^13^C = 2.1 ± 0.2‰ and Δ^15^N = 3.9 ± 0.3‰, respectively. To calculate the mixing space, we considered the digestible elemental concentration of C and N (Supplementary Table S3) to avoid the bias introduced in dietary estimation by differences in stoichiometry and digestibility of food sources^28,128,129^. The efficiency of SIMMs is related to the number of sources and it decreases when the number of sources is greater than the number of tracers/isotopes plus one^130^, or when the isotopic distance of sources in the mixing space is low^130^. Therefore, to improve the SIMMs accuracy and precision, we incorporated “a priori” dietary knowledge on this bear population as informative priors. Specifically, for each season we used as priors the estimated digestible energy content (EDEC%) based on the analysis of 2359 scats collected from the same bear population from June 2006 to December 2009^13^ (Table 2). Before running the models, the adequacy of the mixing spaces (consumers, resources, and TEF data) was evaluated and validated using simulated mixing polygons and all values outside the 95% mixing region were excluded from the models^124^. For all SIMMs models, we ran three Markov Chain Monte Carlo chains of 300,000 iteration each with a burn-in of 200,000 and a thinning rate of 100 iteration. Each model was checked for chains convergence by visual inspection of trace-plot and using the Gelman-Rubin and the Geweke diagnostic test.

Results of mixing models were reported as means of estimated dietary proportion with their associated standard deviations and 95% credible intervals (CI). All statistical analyses and stable isotope mixing models were performed using R version 4.0.3^131^ and the R package MixSIAR^80,127^. Unless otherwise specified, all values in the text were reported as mean values ± standard deviation.

## Supplementary Information

Supplementary Information.
