# Supplementary material for: Gaining insight into the assimilated diet of small bear populations by stable isotope analysis
Source: Sci Rep. 2021 Jul 8;11:14118. doi: 10.1038/s41598-021-93507-y (PMC8266819; doi:10.1038/s41598-021-93507-y)
Supplement: Supplementary file 1 — Supplementary Information. [file 41598_2021_93507_MOESM1_ESM.docx]

**SUPPLEMENTARY INFORMATION**

**Gaining insight on the assimilated diet of small bear populations by stable isotope analysis**

Giulio Careddu^1^, Paolo Ciucci^2,*^, Stella Mondovì^1,2^, Edoardo Calizza^1^, Loreto Rossi^1^, Maria Letizia Costantini^1^

^1^ Department of Environmental Biology, Sapienza University of Rome, Rome, Italy

^2^ Department of Biology and Biotechnologies “Charles Darwin”, Sapienza University of Rome, Rome, Italy

* Corresponding Author: [paolo.ciucci@uniroma1.it](mailto:paolo.ciucci@uniroma1.it)

**Table S1.** Individual mean Carbon and Nitrogen isotopic signatures across hair sections of 27 Apennine bears noninvasively sampled from June−September 2014 in the Abruzzo Lazio and Molise National Park (central Italy).

| **Bear ID** | **Management**  **Status**^a^ | **Sex** | **Sampling period** |  | **δ^13^C** | |  | **δ^15^N** | |  |
| --- | --- | --- | --- | --- | --- | --- | --- | --- | --- | --- |
|  |  |  |  |  | **Mean** | **SD** |  | **Mean** | **SD** | **Notes^c^** |
|  |  |  |  |  |  |  |  |  |  |  |
| 89acc03a | Wild | F | Post moult |  | -22.1 | 0.2 |  | 3.9 | 0.6 | Cub of F05 |
| 89acc04 | Unkn | M | Post moult |  | -22.2 | 0.3 |  | 4.0 | 0.6 |  |
| Acc079 | Mgnt* | F | Post moult |  | -22.4 | 0.4 |  | 2.5 | 0.4 | Sampled by park wardens at a livestock depredation event |
| F05 | Wild | F | Post moult |  | -22.1 | 0.5 |  | 3.2 | 0.5 |  |
| M12 | Wild | M | Pre moult |  | -21.8 | 0.5 |  | -0.4 | 2.1 |  |
|  |  |  | Post moult |  | -21.8 | 0.5 |  | -0.1 | 2.5 |  |
| F09 | Mgnt* | F | Pre moult |  | -21.8 | 0.6 |  | 2.9 | 0.7 | Known to occasionally feed on cultivations |
|  |  |  | Post moult |  | -22.5 | 1.7 |  | 3.7 | 0.8 |  |
| FP01 | Mgnt*** | F | Pre moult |  | -21.5 | 0.5 |  | 5.3 | 0.5 | Known to repeatedly visit villages for anthropogenic foods, in particular poultry |
|  |  |  | Post moult |  | -21.2 | 0.3 |  | 5.4 | 0.5 |  |
| Hs028 | Unkn | F | Post moult |  | -21.8 | 0.1 |  | 2.7 | 1.8 |  |
| Hs0853 | Unkn | F | Pre moult |  | -21.0 | 0.3 |  | -0.5 | 0.6 |  |
| Hs1293 | Unkn | M | Pre moult |  | -22.0 | 0.1 |  | 2.7 | 0.6 |  |
|  | Unkn | M | Post moult |  | -21.9 | 0.4 |  | 3.3 | 0.4 |  |
| Hs330 | Unkn | F | Post moult |  | -22.5 | 0.2 |  | 3.5 | 0.8 |  |
| Hs338 | Mgnt* | M | Pre moult |  | -23.1 | 0.3 |  | 4.4 | 0.5 | Sampled by park wardens at a livestock depredation event |
|  |  |  | Post moult |  | -22.6 | 0.2 |  | 4.3 | 1.1 |  |
| Hs343 | Mgnt* | F | Pre moult |  | -22.3 | 0.4 |  | 2.2 | 2.5 | Sampled by park wardens at a livestock depredation event |
|  |  |  | Post moult |  | -21.8 | 0.7 |  | 3.0 | 0.6 |  |
| Hs349 | Mgnt* | M | Post moult |  | -23.2 | 0.4 |  | 4.2 | 0.4 | Sampled by park wardens at a livestock depredation event |
| Hs374 | Unkn | M | Pre moult |  | -22.1 | 0.5 |  | 2.9 | 1.5 |  |
| Hs465 | Mgnt*** | M | Pre moult |  | -23.5 | 0.3 |  | 4.7 | 0.1 | Sampled by park wardens at a depredation event (poultry and beehives)– genotype compatible with FP01’s son |
| Ram011 | Unkn | F | Post moult |  | -21.6 | 0.7 |  | 2.3 | 1.1 |  |
| Ram0529 | Unkn | F | Post moult |  | -22.8 | 0.7 |  | 2.3 | 1.6 |  |
| F01 | Wild | F | Post moult |  | -23.1 | 0.4 |  | 3.9 | 1.2 |  |
| Rt0429 | Unkn | M | Post moult |  | -21.4 | 0.5 |  | 2.8 | 0.4 |  |
| Rt109 | Unkn | F | Post moult |  | -21.3 | 0.5 |  | 3.4 | 0.5 |  |
| Rt148 | Mgnt** | M | Pre moult |  | -22.4 | 0.2 |  | 4.2 | 0.3 | Sampled by park wardens at a beehives depredaiton event |
| M09 | Mgnt* | M | Post moult |  | -22.7 | 0.3 |  | 3.9 | 0.3 | Sampled by park wardens at a livestock depredation event |
| F13 | Mgnt** | F | Pre moult |  | -22.7 | 0.4 |  | 3.2 | 0.9 | Occasionally sampled by park wardens at damages to agriculture (crops) |
|  |  |  | Post moult |  | -21.8 | 0.2 |  | 3.1 | 0.6 |  |
| F07 (F07) | Mgnt** | F | Pre moult |  | -22.3 | 0.4 |  | 3.5 | 0.5 | Known to occasionally fed on cultivations |
|  |  |  |  |  | -22.2 | 0.3 |  | 3.4 | 0.3 |  |
| F03 (F03) | Wild | F | Pre moult |  | -21.8 | 0.6 |  | 1.9 | 2.0 |  |
| M13 (M13) | Wild | M | Post moult |  | -22.4 | 0.4 |  | 4.4 | 1.2 |  |

**NOTES**

^a^Mgnt = management bear, that is known or suspected to be food-conditioned (***: known to be food-conditioned; **: suspected to be food-conditioned as occasionally sampled by park wardens on beehives and/or crop damage events; *: suspected to be food-conditioned as occasionally sampled by park wardens on livestock damage events); Wild = non-management bear, not food-conditioned or problematic; Unkn = bear of unknown management status.

**Table S2.** Mean and 95% credibility interval estimated posterior proportional dietary contribution, obtained with informative priors, for individual Apennine Brown bears in four trophic seasons.

|  | **Spring** |  |  |  | **Early summer** |  |  |  |  | **Late Summer** |  |  |  |  | **Autumn** |  |  |  |
| --- | --- | --- | --- | --- | --- | --- | --- | --- | --- | --- | --- | --- | --- | --- | --- | --- | --- | --- |
|  | **Mean** | **2.50%** | **97.50%** |  |  | **Mean** | **2.50%** | **97.50%** |  |  | **Mean** | **2.50%** | **97.50%** |  |  | **Mean** | **2.50%** | **97.50%** |
| ACC079. Cultivated fleshy fruits | 0.000 | 0.000 | 0.001 |  | 89ACC03a. Cultivated fleshy fruits | 0.079 | 0.001 | 0.462 |  | 89ACC03a_2. Cultivated fleshy fruits | 0.184 | 0.006 | 0.643 |  | M12. Cultivated fleshy fruits | 0.024 | 0.000 | 0.145 |
| F05. Cultivated fleshy fruits | 0.000 | 0.000 | 0.002 |  | 89ACC04. Cultivated fleshy fruits | 0.074 | 0.001 | 0.413 |  | 89ACC04_2. Cultivated fleshy fruits | 0.192 | 0.007 | 0.704 |  | F09. Cultivated fleshy fruits | 0.262 | 0.000 | 1.000 |
| M12. Cultivated fleshy fruits | 0.000 | 0.000 | 0.001 |  | ACC079. Cultivated fleshy fruits | 0.059 | 0.000 | 0.333 |  | ACC079_2. Cultivated fleshy fruits | 0.149 | 0.005 | 0.557 |  | FP01. Cultivated fleshy fruits | 0.176 | 0.000 | 0.982 |
| F09. Cultivated fleshy fruits | 0.000 | 0.000 | 0.001 |  | F05. Cultivated fleshy fruits | 0.062 | 0.000 | 0.335 |  | F05_2. Cultivated fleshy fruits | 0.153 | 0.004 | 0.652 |  | HS0853. Cultivated fleshy fruits | 0.026 | 0.000 | 0.208 |
| FP01. Cultivated fleshy fruits | 0.000 | 0.000 | 0.002 |  | M12. Cultivated fleshy fruits | 0.025 | 0.000 | 0.124 |  | M12. Cultivated fleshy fruits | 0.039 | 0.001 | 0.167 |  | HS1293. Cultivated fleshy fruits | 0.158 | 0.000 | 0.971 |
| FP01_0. Cultivated fleshy fruits | 0.000 | 0.000 | 0.001 |  | F09. Cultivated fleshy fruits | 0.072 | 0.000 | 0.431 |  | M12_2. Cultivated fleshy fruits | 0.057 | 0.000 | 0.654 |  | HS338. Cultivated fleshy fruits | 0.443 | 0.000 | 1.000 |
| HS028. Cultivated fleshy fruits | 0.000 | 0.000 | 0.001 |  | FP01. Cultivated fleshy fruits | 0.044 | 0.000 | 0.226 |  | F09. Cultivated fleshy fruits | 0.177 | 0.005 | 0.670 |  | HS343. Cultivated fleshy fruits | 0.027 | 0.000 | 0.213 |
| HS1293. Cultivated fleshy fruits | 0.000 | 0.000 | 0.002 |  | HS028. Cultivated fleshy fruits | 0.099 | 0.001 | 0.612 |  | F09_2. Cultivated fleshy fruits | 0.184 | 0.006 | 0.661 |  | HS374. Cultivated fleshy fruits | 0.131 | 0.000 | 0.936 |
| HS330. Cultivated fleshy fruits | 0.000 | 0.000 | 0.001 |  | HS0853. Cultivated fleshy fruits | 0.047 | 0.000 | 0.645 |  | FP01. Cultivated fleshy fruits | 0.177 | 0.009 | 0.562 |  | HS465. Cultivated fleshy fruits | 0.547 | 0.000 | 1.000 |
| HS338. Cultivated fleshy fruits | 0.000 | 0.000 | 0.001 |  | HS1293. Cultivated fleshy fruits | 0.070 | 0.001 | 0.367 |  | FP01_2. Cultivated fleshy fruits | 0.181 | 0.006 | 0.637 |  | RT148. Cultivated fleshy fruits | 0.395 | 0.000 | 1.000 |
| HS343. Cultivated fleshy fruits | 0.000 | 0.000 | 0.001 |  | HS330. Cultivated fleshy fruits | 0.067 | 0.001 | 0.352 |  | HS028_2. Cultivated fleshy fruits | 0.187 | 0.006 | 0.644 |  | F13. Cultivated fleshy fruits | 0.208 | 0.000 | 0.996 |
| HS343_0. Cultivated fleshy fruits | 0.000 | 0.000 | 0.002 |  | HS338. Cultivated fleshy fruits | 0.050 | 0.001 | 0.257 |  | HS0853. Cultivated fleshy fruits | 0.047 | 0.001 | 0.216 |  | F07. Cultivated fleshy fruits | 0.448 | 0.000 | 1.000 |
| HS349. Cultivated fleshy fruits | 0.000 | 0.000 | 0.001 |  | HS343. Cultivated fleshy fruits | 0.094 | 0.001 | 0.646 |  | HS1293. Cultivated fleshy fruits | 0.160 | 0.006 | 0.549 |  | F03. Cultivated fleshy fruits | 0.032 | 0.000 | 0.267 |
| RAM011. Cultivated fleshy fruits | 0.000 | 0.000 | 0.001 |  | HS349. Cultivated fleshy fruits | 0.068 | 0.000 | 0.591 |  | HS1293_2. Cultivated fleshy fruits | 0.168 | 0.007 | 0.589 |  | M12. Formicidae | 0.001 | 0.000 | 0.009 |
| RAM0529. Cultivated fleshy fruits | 0.000 | 0.000 | 0.001 |  | HS374. Cultivated fleshy fruits | 0.066 | 0.000 | 0.407 |  | HS330_2. Cultivated fleshy fruits | 0.185 | 0.006 | 0.694 |  | F09. Formicidae | 0.012 | 0.000 | 0.115 |
| F01. Cultivated fleshy fruits | 0.000 | 0.000 | 0.001 |  | HS465. Cultivated fleshy fruits | 0.081 | 0.000 | 0.596 |  | HS338. Cultivated fleshy fruits | 0.248 | 0.008 | 0.818 |  | FP01. Formicidae | 0.360 | 0.000 | 1.000 |
| RT0429. Cultivated fleshy fruits | 0.000 | 0.000 | 0.001 |  | RAM011. Cultivated fleshy fruits | 0.077 | 0.000 | 0.565 |  | HS338_2. Cultivated fleshy fruits | 0.187 | 0.009 | 0.611 |  | HS0853. Formicidae | 0.001 | 0.000 | 0.013 |
| RT109. Cultivated fleshy fruits | 0.000 | 0.000 | 0.001 |  | RAM0529. Cultivated fleshy fruits | 0.054 | 0.000 | 0.307 |  | HS343. Cultivated fleshy fruits | 0.095 | 0.002 | 0.431 |  | HS1293. Formicidae | 0.013 | 0.000 | 0.113 |
| F13. Cultivated fleshy fruits | 0.000 | 0.000 | 0.001 |  | F01. Cultivated fleshy fruits | 0.063 | 0.000 | 0.411 |  | HS343_2. Cultivated fleshy fruits | 0.178 | 0.005 | 0.662 |  | HS338. Formicidae | 0.026 | 0.000 | 0.274 |
| F13_0. Cultivated fleshy fruits | 0.000 | 0.000 | 0.001 |  | RT0429. Cultivated fleshy fruits | 0.064 | 0.000 | 0.415 |  | HS349_2. Cultivated fleshy fruits | 0.208 | 0.005 | 0.765 |  | HS343. Formicidae | 0.001 | 0.000 | 0.012 |
| F07. Cultivated fleshy fruits | 0.000 | 0.000 | 0.002 |  | RT109. Cultivated fleshy fruits | 0.087 | 0.000 | 0.689 |  | HS374. Cultivated fleshy fruits | 0.178 | 0.005 | 0.689 |  | HS374. Formicidae | 0.008 | 0.000 | 0.077 |
| F07_0. Cultivated fleshy fruits | 0.000 | 0.000 | 0.001 |  | RT148. Cultivated fleshy fruits | 0.074 | 0.001 | 0.479 |  | HS465. Cultivated fleshy fruits | 0.272 | 0.006 | 0.881 |  | HS465. Formicidae | 0.007 | 0.000 | 0.067 |
| F03_0. Cultivated fleshy fruits | 0.000 | 0.000 | 0.001 |  | M09. Cultivated fleshy fruits | 0.071 | 0.001 | 0.427 |  | RAM011_2. Cultivated fleshy fruits | 0.186 | 0.006 | 0.672 |  | RT148. Formicidae | 0.082 | 0.000 | 0.619 |
| M13. Cultivated fleshy fruits | 0.000 | 0.000 | 0.001 |  | F13. Cultivated fleshy fruits | 0.059 | 0.000 | 0.333 |  | RAM0529_2. Cultivated fleshy fruits | 0.206 | 0.006 | 0.752 |  | F13. Formicidae | 0.011 | 0.000 | 0.109 |
| ACC079. Formicidae | 0.040 | 0.002 | 0.153 |  | F07. Cultivated fleshy fruits | 0.067 | 0.001 | 0.324 |  | F01_2. Cultivated fleshy fruits | 0.238 | 0.007 | 0.804 |  | F07. Formicidae | 0.024 | 0.000 | 0.271 |
| F05. Formicidae | 0.070 | 0.003 | 0.268 |  | F03. Cultivated fleshy fruits | 0.099 | 0.000 | 0.695 |  | RT0429_2. Cultivated fleshy fruits | 0.175 | 0.005 | 0.673 |  | F03. Formicidae | 0.002 | 0.000 | 0.021 |
| M12. Formicidae | 0.022 | 0.001 | 0.078 |  | M13. Cultivated fleshy fruits | 0.076 | 0.000 | 0.441 |  | RT109_2. Cultivated fleshy fruits | 0.158 | 0.004 | 0.599 |  | M12. Hard Mast | 0.835 | 0.000 | 1.000 |
| F09. Formicidae | 0.024 | 0.001 | 0.093 |  | 89ACC03a. Formicidae | 0.241 | 0.011 | 0.600 |  | RT148. Cultivated fleshy fruits | 0.177 | 0.008 | 0.571 |  | F09. Hard Mast | 0.258 | 0.000 | 0.996 |
| FP01. Formicidae | 0.108 | 0.003 | 0.646 |  | 89ACC04. Formicidae | 0.232 | 0.010 | 0.566 |  | M09_2. Cultivated fleshy fruits | 0.187 | 0.006 | 0.642 |  | FP01. Hard Mast | 0.201 | 0.000 | 0.836 |
| FP01_0. Formicidae | 0.084 | 0.002 | 0.571 |  | ACC079. Formicidae | 0.096 | 0.003 | 0.280 |  | F13. Cultivated fleshy fruits | 0.203 | 0.006 | 0.737 |  | HS0853. Hard Mast | 0.813 | 0.000 | 1.000 |
| HS028. Formicidae | 0.018 | 0.001 | 0.061 |  | F05. Formicidae | 0.153 | 0.006 | 0.381 |  | F13_2. Cultivated fleshy fruits | 0.184 | 0.005 | 0.680 |  | HS1293. Hard Mast | 0.479 | 0.000 | 1.000 |
| HS1293. Formicidae | 0.062 | 0.002 | 0.251 |  | M12. Formicidae | 0.019 | 0.000 | 0.077 |  | F07. Cultivated fleshy fruits | 0.177 | 0.008 | 0.585 |  | HS338. Hard Mast | 0.073 | 0.000 | 0.611 |
| HS330. Formicidae | 0.040 | 0.002 | 0.153 |  | F09. Formicidae | 0.236 | 0.014 | 0.599 |  | F07_2. Cultivated fleshy fruits | 0.175 | 0.005 | 0.646 |  | HS343. Hard Mast | 0.770 | 0.000 | 1.000 |
| HS338. Formicidae | 0.044 | 0.002 | 0.180 |  | FP01. Formicidae | 0.624 | 0.166 | 0.965 |  | F03. Cultivated fleshy fruits | 0.061 | 0.001 | 0.285 |  | HS374. Hard Mast | 0.464 | 0.000 | 1.000 |
| HS343. Formicidae | 0.032 | 0.001 | 0.124 |  | HS028. Formicidae | 0.243 | 0.007 | 0.751 |  | M13_2. Cultivated fleshy fruits | 0.205 | 0.007 | 0.722 |  | HS465. Hard Mast | 0.029 | 0.000 | 0.207 |
| HS343_0. Formicidae | 0.074 | 0.002 | 0.326 |  | HS0853. Formicidae | 0.034 | 0.000 | 0.145 |  | 89ACC03a_2. Formicidae | 0.064 | 0.001 | 0.303 |  | RT148. Hard Mast | 0.143 | 0.000 | 0.888 |
| HS349. Formicidae | 0.058 | 0.002 | 0.257 |  | HS1293. Formicidae | 0.231 | 0.015 | 0.518 |  | 89ACC04_2. Formicidae | 0.076 | 0.001 | 0.379 |  | F13. Hard Mast | 0.237 | 0.000 | 0.996 |
| RAM011. Formicidae | 0.020 | 0.001 | 0.073 |  | HS330. Formicidae | 0.257 | 0.010 | 0.616 |  | ACC079_2. Formicidae | 0.028 | 0.001 | 0.116 |  | F07. Hard Mast | 0.088 | 0.000 | 0.831 |
| RAM0529. Formicidae | 0.014 | 0.000 | 0.052 |  | HS338. Formicidae | 0.170 | 0.004 | 0.486 |  | F05_2. Formicidae | 0.031 | 0.001 | 0.148 |  | F03. Hard Mast | 0.805 | 0.000 | 1.000 |
| F01. Formicidae | 0.035 | 0.002 | 0.134 |  | HS343. Formicidae | 0.223 | 0.006 | 0.592 |  | M12. Formicidae | 0.007 | 0.000 | 0.028 |  | M12. Herbs | 0.005 | 0.000 | 0.048 |
| RT0429. Formicidae | 0.035 | 0.001 | 0.144 |  | HS349. Formicidae | 0.161 | 0.003 | 0.602 |  | M12_2. Formicidae | 0.008 | 0.000 | 0.034 |  | F09. Herbs | 0.142 | 0.000 | 0.871 |
| RT109. Formicidae | 0.073 | 0.003 | 0.326 |  | HS374. Formicidae | 0.445 | 0.022 | 0.936 |  | F09. Formicidae | 0.049 | 0.001 | 0.211 |  | FP01. Herbs | 0.040 | 0.000 | 0.345 |
| F13. Formicidae | 0.039 | 0.002 | 0.139 |  | HS465. Formicidae | 0.274 | 0.006 | 0.885 |  | F09_2. Formicidae | 0.065 | 0.001 | 0.315 |  | HS0853. Herbs | 0.006 | 0.000 | 0.053 |
| F13_0. Formicidae | 0.049 | 0.002 | 0.187 |  | RAM011. Formicidae | 0.121 | 0.003 | 0.347 |  | FP01. Formicidae | 0.156 | 0.003 | 0.674 |  | HS1293. Herbs | 0.033 | 0.000 | 0.290 |
| F07. Formicidae | 0.061 | 0.003 | 0.239 |  | RAM0529. Formicidae | 0.131 | 0.004 | 0.341 |  | FP01_2. Formicidae | 0.167 | 0.002 | 0.783 |  | HS338. Herbs | 0.287 | 0.000 | 0.855 |
| F07_0. Formicidae | 0.053 | 0.002 | 0.229 |  | F01. Formicidae | 0.209 | 0.005 | 0.656 |  | HS028_2. Formicidae | 0.096 | 0.002 | 0.479 |  | HS343. Herbs | 0.011 | 0.000 | 0.112 |
| F03_0. Formicidae | 0.051 | 0.002 | 0.195 |  | RT0429. Formicidae | 0.103 | 0.002 | 0.309 |  | HS0853. Formicidae | 0.008 | 0.000 | 0.034 |  | HS374. Herbs | 0.042 | 0.000 | 0.385 |
| M13. Formicidae | 0.041 | 0.001 | 0.180 |  | RT109. Formicidae | 0.216 | 0.004 | 0.740 |  | HS1293. Formicidae | 0.038 | 0.001 | 0.154 |  | HS465. Herbs | 0.306 | 0.000 | 0.907 |
| ACC079. Hard Mast | 0.415 | 0.059 | 0.839 |  | RT148. Formicidae | 0.245 | 0.008 | 0.725 |  | HS1293_2. Formicidae | 0.040 | 0.001 | 0.163 |  | RT148. Herbs | 0.151 | 0.000 | 0.757 |
| F05. Hard Mast | 0.421 | 0.106 | 0.773 |  | M09. Formicidae | 0.275 | 0.008 | 0.805 |  | HS330_2. Formicidae | 0.054 | 0.001 | 0.260 |  | F13. Herbs | 0.197 | 0.000 | 0.945 |
| M12. Hard Mast | 0.456 | 0.028 | 0.920 |  | F13. Formicidae | 0.186 | 0.009 | 0.426 |  | HS338. Formicidae | 0.053 | 0.001 | 0.256 |  | F07. Herbs | 0.299 | 0.000 | 0.914 |
| F09. Hard Mast | 0.123 | 0.004 | 0.772 |  | F07. Formicidae | 0.190 | 0.011 | 0.418 |  | HS338_2. Formicidae | 0.095 | 0.003 | 0.435 |  | F03. Herbs | 0.006 | 0.000 | 0.060 |
| FP01. Hard Mast | 0.459 | 0.050 | 0.836 |  | F03. Formicidae | 0.156 | 0.003 | 0.462 |  | HS343. Formicidae | 0.015 | 0.000 | 0.062 |  | M12. Ungulates | 0.001 | 0.000 | 0.006 |
| FP01_0. Hard Mast | 0.526 | 0.050 | 0.879 |  | M13. Formicidae | 0.348 | 0.012 | 0.858 |  | HS343_2. Formicidae | 0.039 | 0.001 | 0.159 |  | F09. Ungulates | 0.006 | 0.000 | 0.045 |
| HS028. Hard Mast | 0.542 | 0.073 | 0.919 |  | 89ACC03a. Hard Mast | 0.125 | 0.001 | 0.640 |  | HS349_2. Formicidae | 0.049 | 0.001 | 0.214 |  | FP01. Ungulates | 0.058 | 0.000 | 0.275 |
| HS1293. Hard Mast | 0.441 | 0.068 | 0.838 |  | 89ACC04. Hard Mast | 0.138 | 0.001 | 0.659 |  | HS374. Formicidae | 0.037 | 0.001 | 0.153 |  | HS0853. Ungulates | 0.001 | 0.000 | 0.010 |
| HS330. Hard Mast | 0.330 | 0.030 | 0.768 |  | ACC079. Hard Mast | 0.099 | 0.001 | 0.527 |  | HS465. Formicidae | 0.042 | 0.001 | 0.199 |  | HS1293. Ungulates | 0.006 | 0.000 | 0.040 |
| HS338. Hard Mast | 0.410 | 0.026 | 0.846 |  | F05. Hard Mast | 0.092 | 0.001 | 0.443 |  | RAM011_2. Formicidae | 0.048 | 0.001 | 0.228 |  | HS338. Ungulates | 0.040 | 0.000 | 0.194 |
| HS343. Hard Mast | 0.386 | 0.031 | 0.863 |  | M12. Hard Mast | 0.170 | 0.000 | 0.968 |  | RAM0529_2. Formicidae | 0.049 | 0.001 | 0.211 |  | HS343. Ungulates | 0.001 | 0.000 | 0.007 |
| HS343_0. Hard Mast | 0.356 | 0.046 | 0.735 |  | F09. Hard Mast | 0.186 | 0.001 | 0.772 |  | F01_2. Formicidae | 0.062 | 0.001 | 0.328 |  | HS374. Ungulates | 0.004 | 0.000 | 0.028 |
| HS349. Hard Mast | 0.244 | 0.021 | 0.632 |  | FP01. Hard Mast | 0.072 | 0.000 | 0.326 |  | RT0429_2. Formicidae | 0.048 | 0.001 | 0.214 |  | HS465. Ungulates | 0.029 | 0.000 | 0.182 |
| RAM011. Hard Mast | 0.673 | 0.092 | 0.961 |  | HS028. Hard Mast | 0.159 | 0.001 | 0.735 |  | RT109_2. Formicidae | 0.036 | 0.001 | 0.152 |  | RT148. Ungulates | 0.037 | 0.000 | 0.193 |
| RAM0529. Hard Mast | 0.488 | 0.018 | 0.948 |  | HS0853. Hard Mast | 0.144 | 0.000 | 0.951 |  | RT148. Formicidae | 0.077 | 0.002 | 0.318 |  | F13. Ungulates | 0.006 | 0.000 | 0.040 |
| F01. Hard Mast | 0.159 | 0.009 | 0.504 |  | HS1293. Hard Mast | 0.156 | 0.002 | 0.654 |  | M09_2. Formicidae | 0.051 | 0.001 | 0.216 |  | F07. Ungulates | 0.024 | 0.000 | 0.132 |
| RT0429. Hard Mast | 0.603 | 0.080 | 0.942 |  | HS330. Hard Mast | 0.098 | 0.001 | 0.474 |  | F13. Formicidae | 0.057 | 0.001 | 0.243 |  | F03. Ungulates | 0.001 | 0.000 | 0.012 |
| RT109. Hard Mast | 0.442 | 0.075 | 0.831 |  | HS338. Hard Mast | 0.055 | 0.001 | 0.242 |  | F13_2. Formicidae | 0.060 | 0.001 | 0.293 |  | M12. Cultivated vegetables | 0.001 | 0.000 | 0.006 |
| F13. Hard Mast | 0.423 | 0.073 | 0.820 |  | HS343. Hard Mast | 0.106 | 0.001 | 0.631 |  | F07. Formicidae | 0.066 | 0.002 | 0.280 |  | F09. Cultivated vegetables | 0.007 | 0.000 | 0.058 |
| F13_0. Hard Mast | 0.313 | 0.030 | 0.716 |  | HS349. Hard Mast | 0.055 | 0.001 | 0.281 |  | F07_2. Formicidae | 0.043 | 0.001 | 0.190 |  | FP01. Cultivated vegetables | 0.025 | 0.000 | 0.202 |
| F07. Hard Mast | 0.346 | 0.055 | 0.703 |  | HS374. Hard Mast | 0.105 | 0.001 | 0.560 |  | F03. Formicidae | 0.010 | 0.000 | 0.041 |  | HS0853. Cultivated vegetables | 0.001 | 0.000 | 0.009 |
| F07_0. Hard Mast | 0.364 | 0.032 | 0.815 |  | HS465. Hard Mast | 0.081 | 0.001 | 0.496 |  | M13_2. Formicidae | 0.081 | 0.002 | 0.442 |  | HS1293. Cultivated vegetables | 0.005 | 0.000 | 0.048 |
| F03_0. Hard Mast | 0.409 | 0.081 | 0.793 |  | RAM011. Hard Mast | 0.157 | 0.001 | 0.831 |  | 89ACC03a_2. Hard Mast | 0.165 | 0.004 | 0.624 |  | HS338. Cultivated vegetables | 0.047 | 0.000 | 0.289 |
| M13. Hard Mast | 0.367 | 0.031 | 0.848 |  | RAM0529. Hard Mast | 0.065 | 0.001 | 0.327 |  | 89ACC04_2. Hard Mast | 0.158 | 0.005 | 0.620 |  | HS343. Cultivated vegetables | 0.001 | 0.000 | 0.007 |
| ACC079. Herbs | 0.330 | 0.029 | 0.708 |  | F01. Hard Mast | 0.065 | 0.001 | 0.340 |  | ACC079_2. Hard Mast | 0.143 | 0.003 | 0.616 |  | HS374. Cultivated vegetables | 0.004 | 0.000 | 0.036 |
| F05. Herbs | 0.273 | 0.040 | 0.553 |  | RT0429. Hard Mast | 0.179 | 0.001 | 0.847 |  | F05_2. Hard Mast | 0.151 | 0.003 | 0.676 |  | HS465. Cultivated vegetables | 0.052 | 0.000 | 0.297 |
| M12. Herbs | 0.338 | 0.019 | 0.834 |  | RT109. Hard Mast | 0.180 | 0.001 | 0.869 |  | M12. Hard Mast | 0.055 | 0.000 | 0.638 |  | RT148. Cultivated vegetables | 0.023 | 0.000 | 0.177 |
| F09. Herbs | 0.733 | 0.057 | 0.951 |  | RT148. Hard Mast | 0.097 | 0.001 | 0.512 |  | M12_2. Hard Mast | 0.065 | 0.000 | 0.803 |  | F13. Cultivated vegetables | 0.006 | 0.000 | 0.050 |
| FP01. Herbs | 0.176 | 0.011 | 0.489 |  | M09. Hard Mast | 0.099 | 0.001 | 0.529 |  | F09. Hard Mast | 0.183 | 0.005 | 0.744 |  | F07. Cultivated vegetables | 0.021 | 0.000 | 0.190 |
| FP01_0. Herbs | 0.155 | 0.007 | 0.519 |  | F13. Hard Mast | 0.080 | 0.001 | 0.393 |  | F09_2. Hard Mast | 0.168 | 0.005 | 0.644 |  | F03. Cultivated vegetables | 0.001 | 0.000 | 0.011 |
| HS028. Herbs | 0.270 | 0.015 | 0.699 |  | F07. Hard Mast | 0.156 | 0.002 | 0.592 |  | FP01. Hard Mast | 0.153 | 0.007 | 0.502 |  | M12. Wild flesy fruits | 0.134 | 0.000 | 1.000 |
| HS1293. Herbs | 0.263 | 0.022 | 0.611 |  | F03. Hard Mast | 0.104 | 0.001 | 0.613 |  | FP01_2. Hard Mast | 0.138 | 0.005 | 0.545 |  | F09. Wild flesy fruits | 0.313 | 0.000 | 1.000 |
| HS330. Herbs | 0.425 | 0.061 | 0.799 |  | M13. Hard Mast | 0.095 | 0.001 | 0.450 |  | HS028_2. Hard Mast | 0.158 | 0.005 | 0.574 |  | FP01. Wild flesy fruits | 0.141 | 0.000 | 0.816 |
| HS338. Herbs | 0.303 | 0.025 | 0.743 |  | 89ACC03a. Herbs | 0.261 | 0.006 | 0.621 |  | HS0853. Hard Mast | 0.056 | 0.001 | 0.541 |  | HS0853. Wild flesy fruits | 0.151 | 0.000 | 1.000 |
| HS343. Herbs | 0.392 | 0.025 | 0.846 |  | 89ACC04. Herbs | 0.266 | 0.005 | 0.635 |  | HS1293. Hard Mast | 0.182 | 0.005 | 0.667 |  | HS1293. Wild flesy fruits | 0.304 | 0.000 | 1.000 |
| HS343_0. Herbs | 0.315 | 0.046 | 0.636 |  | ACC079. Herbs | 0.487 | 0.017 | 0.872 |  | HS1293_2. Hard Mast | 0.175 | 0.005 | 0.647 |  | HS338. Wild flesy fruits | 0.085 | 0.000 | 0.824 |
| HS349. Herbs | 0.463 | 0.112 | 0.775 |  | F05. Herbs | 0.446 | 0.019 | 0.780 |  | HS330_2. Hard Mast | 0.163 | 0.003 | 0.646 |  | HS343. Wild flesy fruits | 0.189 | 0.000 | 1.000 |
| RAM011. Herbs | 0.168 | 0.004 | 0.636 |  | M12. Herbs | 0.105 | 0.000 | 0.869 |  | HS338. Hard Mast | 0.117 | 0.004 | 0.580 |  | HS374. Wild flesy fruits | 0.347 | 0.000 | 1.000 |
| RAM0529. Herbs | 0.359 | 0.009 | 0.914 |  | F09. Herbs | 0.190 | 0.003 | 0.543 |  | HS338_2. Hard Mast | 0.163 | 0.006 | 0.584 |  | HS465. Wild flesy fruits | 0.030 | 0.000 | 0.230 |
| F01. Herbs | 0.640 | 0.198 | 0.880 |  | FP01. Herbs | 0.082 | 0.001 | 0.279 |  | HS343. Hard Mast | 0.111 | 0.001 | 0.593 |  | RT148. Wild flesy fruits | 0.170 | 0.000 | 0.896 |
| RT0429. Herbs | 0.183 | 0.006 | 0.635 |  | HS028. Herbs | 0.143 | 0.002 | 0.484 |  | HS343_2. Hard Mast | 0.155 | 0.004 | 0.670 |  | F13. Wild flesy fruits | 0.336 | 0.000 | 1.000 |
| RT109. Herbs | 0.246 | 0.019 | 0.598 |  | HS0853. Herbs | 0.223 | 0.000 | 0.947 |  | HS349_2. Hard Mast | 0.138 | 0.004 | 0.663 |  | F07. Wild flesy fruits | 0.096 | 0.000 | 0.909 |
| F13. Herbs | 0.323 | 0.037 | 0.670 |  | HS1293. Herbs | 0.261 | 0.005 | 0.584 |  | HS374. Hard Mast | 0.148 | 0.003 | 0.681 |  | F03. Wild flesy fruits | 0.152 | 0.000 | 1.000 |
| F13_0. Herbs | 0.425 | 0.085 | 0.780 |  | HS330. Herbs | 0.324 | 0.021 | 0.674 |  | HS465. Hard Mast | 0.108 | 0.003 | 0.602 |  |  |  |  |  |
| F07. Herbs | 0.353 | 0.067 | 0.656 |  | HS338. Herbs | 0.504 | 0.123 | 0.783 |  | RAM011_2. Hard Mast | 0.159 | 0.004 | 0.647 |  |  |  |  |  |
| F07_0. Herbs | 0.353 | 0.034 | 0.757 |  | HS343. Herbs | 0.248 | 0.003 | 0.636 |  | RAM0529_2. Hard Mast | 0.132 | 0.004 | 0.627 |  |  |  |  |  |
| F03_0. Herbs | 0.312 | 0.040 | 0.636 |  | HS349. Herbs | 0.475 | 0.009 | 0.821 |  | F01_2. Hard Mast | 0.127 | 0.004 | 0.578 |  |  |  |  |  |
| M13. Herbs | 0.388 | 0.033 | 0.811 |  | HS374. Herbs | 0.145 | 0.002 | 0.502 |  | RT0429_2. Hard Mast | 0.163 | 0.004 | 0.679 |  |  |  |  |  |
| ACC079. Ungulates | 0.030 | 0.003 | 0.074 |  | HS465. Herbs | 0.289 | 0.004 | 0.731 |  | RT109_2. Hard Mast | 0.175 | 0.004 | 0.692 |  |  |  |  |  |
| F05. Ungulates | 0.044 | 0.005 | 0.093 |  | RAM011. Herbs | 0.272 | 0.002 | 0.775 |  | RT148. Hard Mast | 0.175 | 0.006 | 0.613 |  |  |  |  |  |
| M12. Ungulates | 0.017 | 0.002 | 0.044 |  | RAM0529. Herbs | 0.540 | 0.050 | 0.857 |  | M09_2. Hard Mast | 0.150 | 0.004 | 0.598 |  |  |  |  |  |
| F09. Ungulates | 0.040 | 0.003 | 0.098 |  | F01. Herbs | 0.412 | 0.016 | 0.764 |  | F13. Hard Mast | 0.140 | 0.004 | 0.580 |  |  |  |  |  |
| FP01. Ungulates | 0.090 | 0.006 | 0.206 |  | RT0429. Herbs | 0.288 | 0.002 | 0.793 |  | F13_2. Hard Mast | 0.170 | 0.004 | 0.666 |  |  |  |  |  |
| FP01_0. Ungulates | 0.080 | 0.006 | 0.195 |  | RT109. Herbs | 0.157 | 0.001 | 0.588 |  | F07. Hard Mast | 0.182 | 0.007 | 0.618 |  |  |  |  |  |
| HS028. Ungulates | 0.012 | 0.001 | 0.032 |  | RT148. Herbs | 0.310 | 0.005 | 0.724 |  | F07_2. Hard Mast | 0.155 | 0.003 | 0.647 |  |  |  |  |  |
| HS1293. Ungulates | 0.046 | 0.005 | 0.107 |  | M09. Herbs | 0.283 | 0.005 | 0.723 |  | F03. Hard Mast | 0.073 | 0.001 | 0.518 |  |  |  |  |  |
| HS330. Ungulates | 0.033 | 0.004 | 0.079 |  | F13. Herbs | 0.444 | 0.033 | 0.764 |  | M13_2. Hard Mast | 0.144 | 0.005 | 0.584 |  |  |  |  |  |
| HS338. Ungulates | 0.048 | 0.006 | 0.116 |  | F07. Herbs | 0.310 | 0.016 | 0.617 |  | 89ACC03a_2. Herbs | 0.081 | 0.001 | 0.379 |  |  |  |  |  |
| HS343. Ungulates | 0.026 | 0.002 | 0.071 |  | F03. Herbs | 0.313 | 0.004 | 0.758 |  | 89ACC04_2. Herbs | 0.081 | 0.001 | 0.385 |  |  |  |  |  |
| HS343_0. Ungulates | 0.061 | 0.008 | 0.136 |  | M13. Herbs | 0.203 | 0.004 | 0.561 |  | ACC079_2. Herbs | 0.135 | 0.001 | 0.632 |  |  |  |  |  |
| HS349. Ungulates | 0.070 | 0.012 | 0.148 |  | 89ACC03a. Ungulates | 0.026 | 0.001 | 0.097 |  | F05_2. Herbs | 0.079 | 0.001 | 0.479 |  |  |  |  |  |
| RAM011. Ungulates | 0.016 | 0.001 | 0.044 |  | 89ACC04. Ungulates | 0.023 | 0.001 | 0.085 |  | M12. Herbs | 0.017 | 0.000 | 0.092 |  |  |  |  |  |
| RAM0529. Ungulates | 0.010 | 0.001 | 0.030 |  | ACC079. Ungulates | 0.014 | 0.000 | 0.052 |  | M12_2. Herbs | 0.017 | 0.000 | 0.083 |  |  |  |  |  |
| F01. Ungulates | 0.052 | 0.009 | 0.106 |  | F05. Ungulates | 0.022 | 0.001 | 0.079 |  | F09. Herbs | 0.060 | 0.001 | 0.285 |  |  |  |  |  |
| RT0429. Ungulates | 0.033 | 0.003 | 0.086 |  | M12. Ungulates | 0.004 | 0.000 | 0.015 |  | F09_2. Herbs | 0.079 | 0.002 | 0.366 |  |  |  |  |  |
| RT109. Ungulates | 0.056 | 0.006 | 0.132 |  | F09. Ungulates | 0.019 | 0.000 | 0.069 |  | FP01. Herbs | 0.061 | 0.002 | 0.251 |  |  |  |  |  |
| F13. Ungulates | 0.027 | 0.003 | 0.065 |  | FP01. Ungulates | 0.044 | 0.000 | 0.160 |  | FP01_2. Herbs | 0.047 | 0.001 | 0.197 |  |  |  |  |  |
| F13_0. Ungulates | 0.039 | 0.004 | 0.090 |  | HS028. Ungulates | 0.028 | 0.001 | 0.106 |  | HS028_2. Herbs | 0.075 | 0.002 | 0.322 |  |  |  |  |  |
| F07. Ungulates | 0.045 | 0.005 | 0.098 |  | HS0853. Ungulates | 0.006 | 0.000 | 0.028 |  | HS0853. Herbs | 0.020 | 0.000 | 0.111 |  |  |  |  |  |
| F07_0. Ungulates | 0.048 | 0.004 | 0.125 |  | HS1293. Ungulates | 0.019 | 0.001 | 0.066 |  | HS1293. Herbs | 0.099 | 0.002 | 0.440 |  |  |  |  |  |
| F03_0. Ungulates | 0.038 | 0.005 | 0.083 |  | HS330. Ungulates | 0.032 | 0.001 | 0.120 |  | HS1293_2. Herbs | 0.104 | 0.001 | 0.489 |  |  |  |  |  |
| M13. Ungulates | 0.035 | 0.003 | 0.093 |  | HS338. Ungulates | 0.051 | 0.001 | 0.144 |  | HS330_2. Herbs | 0.091 | 0.001 | 0.442 |  |  |  |  |  |
| ACC079. Cultivated vegetables | 0.011 | 0.000 | 0.050 |  | HS343. Ungulates | 0.027 | 0.000 | 0.093 |  | HS338. Herbs | 0.099 | 0.002 | 0.540 |  |  |  |  |  |
| F05. Cultivated vegetables | 0.014 | 0.000 | 0.061 |  | HS349. Ungulates | 0.052 | 0.001 | 0.168 |  | HS338_2. Herbs | 0.099 | 0.002 | 0.405 |  |  |  |  |  |
| M12. Cultivated vegetables | 0.007 | 0.000 | 0.031 |  | HS374. Ungulates | 0.034 | 0.000 | 0.148 |  | HS343. Herbs | 0.086 | 0.001 | 0.563 |  |  |  |  |  |
| F09. Cultivated vegetables | 0.010 | 0.000 | 0.049 |  | HS465. Ungulates | 0.047 | 0.001 | 0.182 |  | HS343_2. Herbs | 0.081 | 0.001 | 0.430 |  |  |  |  |  |
| FP01. Cultivated vegetables | 0.016 | 0.000 | 0.073 |  | RAM011. Ungulates | 0.016 | 0.000 | 0.060 |  | HS349_2. Herbs | 0.084 | 0.001 | 0.472 |  |  |  |  |  |
| FP01_0. Cultivated vegetables | 0.013 | 0.000 | 0.059 |  | RAM0529. Ungulates | 0.023 | 0.000 | 0.081 |  | HS374. Herbs | 0.081 | 0.001 | 0.420 |  |  |  |  |  |
| HS028. Cultivated vegetables | 0.006 | 0.000 | 0.024 |  | F01. Ungulates | 0.052 | 0.001 | 0.172 |  | HS465. Herbs | 0.086 | 0.001 | 0.569 |  |  |  |  |  |
| HS1293. Cultivated vegetables | 0.014 | 0.000 | 0.057 |  | RT0429. Ungulates | 0.013 | 0.000 | 0.049 |  | RAM011_2. Herbs | 0.089 | 0.001 | 0.434 |  |  |  |  |  |
| HS330. Cultivated vegetables | 0.013 | 0.000 | 0.057 |  | RT109. Ungulates | 0.019 | 0.000 | 0.073 |  | RAM0529_2. Herbs | 0.111 | 0.001 | 0.558 |  |  |  |  |  |
| HS338. Cultivated vegetables | 0.016 | 0.000 | 0.079 |  | RT148. Ungulates | 0.032 | 0.000 | 0.116 |  | F01_2. Herbs | 0.068 | 0.001 | 0.348 |  |  |  |  |  |
| HS343. Cultivated vegetables | 0.010 | 0.000 | 0.042 |  | M09. Ungulates | 0.035 | 0.001 | 0.139 |  | RT0429_2. Herbs | 0.084 | 0.001 | 0.410 |  |  |  |  |  |
| HS343_0. Cultivated vegetables | 0.018 | 0.000 | 0.083 |  | F13. Ungulates | 0.025 | 0.000 | 0.085 |  | RT109_2. Herbs | 0.073 | 0.001 | 0.394 |  |  |  |  |  |
| HS349. Cultivated vegetables | 0.019 | 0.000 | 0.102 |  | F07. Ungulates | 0.018 | 0.001 | 0.063 |  | RT148. Herbs | 0.102 | 0.002 | 0.428 |  |  |  |  |  |
| RAM011. Cultivated vegetables | 0.006 | 0.000 | 0.028 |  | F03. Ungulates | 0.029 | 0.001 | 0.104 |  | M09_2. Herbs | 0.120 | 0.002 | 0.529 |  |  |  |  |  |
| RAM0529. Cultivated vegetables | 0.005 | 0.000 | 0.020 |  | M13. Ungulates | 0.044 | 0.000 | 0.157 |  | F13. Herbs | 0.106 | 0.001 | 0.504 |  |  |  |  |  |
| F01. Cultivated vegetables | 0.016 | 0.000 | 0.084 |  | 89ACC03a. Cultivated vegetables | 0.019 | 0.000 | 0.096 |  | F13_2. Herbs | 0.065 | 0.001 | 0.312 |  |  |  |  |  |
| RT0429. Cultivated vegetables | 0.010 | 0.000 | 0.042 |  | 89ACC04. Cultivated vegetables | 0.018 | 0.000 | 0.089 |  | F07. Herbs | 0.100 | 0.002 | 0.437 |  |  |  |  |  |
| RT109. Cultivated vegetables | 0.015 | 0.001 | 0.065 |  | ACC079. Cultivated vegetables | 0.014 | 0.000 | 0.067 |  | F07_2. Herbs | 0.093 | 0.001 | 0.482 |  |  |  |  |  |
| F13. Cultivated vegetables | 0.011 | 0.000 | 0.046 |  | F05. Cultivated vegetables | 0.019 | 0.000 | 0.093 |  | F03. Herbs | 0.029 | 0.000 | 0.162 |  |  |  |  |  |
| F13_0. Cultivated vegetables | 0.014 | 0.000 | 0.063 |  | M12. Cultivated vegetables | 0.003 | 0.000 | 0.017 |  | M13_2. Herbs | 0.074 | 0.002 | 0.359 |  |  |  |  |  |
| F07. Cultivated vegetables | 0.015 | 0.000 | 0.069 |  | F09. Cultivated vegetables | 0.013 | 0.000 | 0.064 |  | 89ACC03a_2. Ungulates | 0.030 | 0.001 | 0.112 |  |  |  |  |  |
| F07_0. Cultivated vegetables | 0.015 | 0.000 | 0.074 |  | FP01. Cultivated vegetables | 0.018 | 0.000 | 0.097 |  | 89ACC04_2. Ungulates | 0.036 | 0.001 | 0.133 |  |  |  |  |  |
| F03_0. Cultivated vegetables | 0.013 | 0.000 | 0.057 |  | HS028. Cultivated vegetables | 0.019 | 0.000 | 0.095 |  | ACC079_2. Ungulates | 0.013 | 0.001 | 0.043 |  |  |  |  |  |
| M13. Cultivated vegetables | 0.012 | 0.000 | 0.056 |  | HS0853. Cultivated vegetables | 0.005 | 0.000 | 0.027 |  | F05_2. Ungulates | 0.014 | 0.000 | 0.054 |  |  |  |  |  |
| ACC079. Wild flesy fruits | 0.173 | 0.009 | 0.609 |  | HS1293. Cultivated vegetables | 0.015 | 0.000 | 0.070 |  | M12. Ungulates | 0.003 | 0.000 | 0.013 |  |  |  |  |  |
| F05. Wild flesy fruits | 0.178 | 0.011 | 0.505 |  | HS330. Cultivated vegetables | 0.026 | 0.000 | 0.131 |  | M12_2. Ungulates | 0.004 | 0.000 | 0.016 |  |  |  |  |  |
| M12. Wild flesy fruits | 0.159 | 0.004 | 0.763 |  | HS338. Cultivated vegetables | 0.044 | 0.000 | 0.208 |  | F09. Ungulates | 0.020 | 0.001 | 0.067 |  |  |  |  |  |
| F09. Wild flesy fruits | 0.070 | 0.002 | 0.590 |  | HS343. Cultivated vegetables | 0.018 | 0.000 | 0.089 |  | F09_2. Ungulates | 0.030 | 0.001 | 0.109 |  |  |  |  |  |
| FP01. Wild flesy fruits | 0.151 | 0.007 | 0.553 |  | HS349. Cultivated vegetables | 0.038 | 0.000 | 0.210 |  | FP01. Ungulates | 0.082 | 0.002 | 0.246 |  |  |  |  |  |
| FP01_0. Wild flesy fruits | 0.140 | 0.005 | 0.601 |  | HS374. Cultivated vegetables | 0.019 | 0.000 | 0.106 |  | FP01_2. Ungulates | 0.103 | 0.002 | 0.323 |  |  |  |  |  |
| HS028. Wild flesy fruits | 0.153 | 0.005 | 0.659 |  | HS465. Cultivated vegetables | 0.031 | 0.000 | 0.181 |  | HS028_2. Ungulates | 0.042 | 0.001 | 0.155 |  |  |  |  |  |
| HS1293. Wild flesy fruits | 0.174 | 0.009 | 0.601 |  | RAM011. Cultivated vegetables | 0.012 | 0.000 | 0.059 |  | HS0853. Ungulates | 0.004 | 0.000 | 0.016 |  |  |  |  |  |
| HS330. Wild flesy fruits | 0.159 | 0.007 | 0.571 |  | RAM0529. Cultivated vegetables | 0.019 | 0.000 | 0.102 |  | HS1293. Ungulates | 0.016 | 0.001 | 0.052 |  |  |  |  |  |
| HS338. Wild flesy fruits | 0.179 | 0.007 | 0.735 |  | F01. Cultivated vegetables | 0.041 | 0.000 | 0.216 |  | HS1293_2. Ungulates | 0.017 | 0.001 | 0.056 |  |  |  |  |  |
| HS343. Wild flesy fruits | 0.154 | 0.007 | 0.613 |  | RT0429. Cultivated vegetables | 0.011 | 0.000 | 0.051 |  | HS330_2. Ungulates | 0.026 | 0.001 | 0.097 |  |  |  |  |  |
| HS343_0. Wild flesy fruits | 0.176 | 0.011 | 0.564 |  | RT109. Cultivated vegetables | 0.012 | 0.000 | 0.059 |  | HS338. Ungulates | 0.040 | 0.001 | 0.139 |  |  |  |  |  |
| HS349. Wild flesy fruits | 0.146 | 0.008 | 0.502 |  | RT148. Cultivated vegetables | 0.024 | 0.000 | 0.123 |  | HS338_2. Ungulates | 0.046 | 0.002 | 0.136 |  |  |  |  |  |
| RAM011. Wild flesy fruits | 0.116 | 0.002 | 0.662 |  | M09. Cultivated vegetables | 0.025 | 0.000 | 0.138 |  | HS343. Ungulates | 0.007 | 0.000 | 0.026 |  |  |  |  |  |
| RAM0529. Wild flesy fruits | 0.125 | 0.002 | 0.735 |  | F13. Cultivated vegetables | 0.018 | 0.000 | 0.089 |  | HS343_2. Ungulates | 0.019 | 0.001 | 0.060 |  |  |  |  |  |
| F01. Wild flesy fruits | 0.097 | 0.004 | 0.407 |  | F07. Cultivated vegetables | 0.015 | 0.000 | 0.073 |  | HS349_2. Ungulates | 0.029 | 0.001 | 0.101 |  |  |  |  |  |
| RT0429. Wild flesy fruits | 0.136 | 0.004 | 0.661 |  | F03. Cultivated vegetables | 0.024 | 0.000 | 0.129 |  | HS374. Ungulates | 0.018 | 0.001 | 0.059 |  |  |  |  |  |
| RT109. Wild flesy fruits | 0.168 | 0.009 | 0.556 |  | M13. Cultivated vegetables | 0.029 | 0.000 | 0.151 |  | HS465. Ungulates | 0.036 | 0.001 | 0.123 |  |  |  |  |  |
| F13. Wild flesy fruits | 0.177 | 0.008 | 0.576 |  | 89ACC03a. Wild flesy fruits | 0.250 | 0.003 | 0.762 |  | RAM011_2. Ungulates | 0.025 | 0.001 | 0.091 |  |  |  |  |  |
| F13_0. Wild flesy fruits | 0.160 | 0.009 | 0.537 |  | 89ACC04. Wild flesy fruits | 0.249 | 0.004 | 0.760 |  | RAM0529_2. Ungulates | 0.029 | 0.001 | 0.095 |  |  |  |  |  |
| F07. Wild flesy fruits | 0.179 | 0.009 | 0.542 |  | ACC079. Wild flesy fruits | 0.231 | 0.003 | 0.823 |  | F01_2. Ungulates | 0.049 | 0.001 | 0.182 |  |  |  |  |  |
| F07_0. Wild flesy fruits | 0.166 | 0.006 | 0.616 |  | F05. Wild flesy fruits | 0.206 | 0.003 | 0.747 |  | RT0429_2. Ungulates | 0.020 | 0.001 | 0.075 |  |  |  |  |  |
| F03_0. Wild flesy fruits | 0.177 | 0.011 | 0.558 |  | M12. Wild flesy fruits | 0.673 | 0.002 | 0.993 |  | RT109_2. Ungulates | 0.016 | 0.001 | 0.051 |  |  |  |  |  |
| M13. Wild flesy fruits | 0.157 | 0.006 | 0.589 |  | F09. Wild flesy fruits | 0.284 | 0.004 | 0.781 |  | RT148. Ungulates | 0.032 | 0.001 | 0.106 |  |  |  |  |  |
|  |  |  |  |  | FP01. Wild flesy fruits | 0.115 | 0.001 | 0.416 |  | M09_2. Ungulates | 0.026 | 0.001 | 0.086 |  |  |  |  |  |
|  |  |  |  |  | HS028. Wild flesy fruits | 0.309 | 0.004 | 0.814 |  | F13. Ungulates | 0.032 | 0.001 | 0.107 |  |  |  |  |  |
|  |  |  |  |  | HS0853. Wild flesy fruits | 0.541 | 0.002 | 0.988 |  | F13_2. Ungulates | 0.027 | 0.001 | 0.102 |  |  |  |  |  |
|  |  |  |  |  | HS1293. Wild flesy fruits | 0.248 | 0.006 | 0.725 |  | F07. Ungulates | 0.027 | 0.001 | 0.088 |  |  |  |  |  |
|  |  |  |  |  | HS330. Wild flesy fruits | 0.196 | 0.003 | 0.674 |  | F07_2. Ungulates | 0.020 | 0.001 | 0.072 |  |  |  |  |  |
|  |  |  |  |  | HS338. Wild flesy fruits | 0.126 | 0.002 | 0.519 |  | F03. Ungulates | 0.004 | 0.000 | 0.017 |  |  |  |  |  |
|  |  |  |  |  | HS343. Wild flesy fruits | 0.285 | 0.003 | 0.822 |  | M13_2. Ungulates | 0.055 | 0.002 | 0.206 |  |  |  |  |  |
|  |  |  |  |  | HS349. Wild flesy fruits | 0.151 | 0.001 | 0.754 |  | 89ACC03a_2. Cultivated vegetables | 0.018 | 0.000 | 0.095 |  |  |  |  |  |
|  |  |  |  |  | HS374. Wild flesy fruits | 0.185 | 0.002 | 0.684 |  | 89ACC04_2. Cultivated vegetables | 0.022 | 0.000 | 0.119 |  |  |  |  |  |
|  |  |  |  |  | HS465. Wild flesy fruits | 0.196 | 0.002 | 0.761 |  | ACC079_2. Cultivated vegetables | 0.011 | 0.000 | 0.050 |  |  |  |  |  |
|  |  |  |  |  | RAM011. Wild flesy fruits | 0.344 | 0.003 | 0.917 |  | F05_2. Cultivated vegetables | 0.010 | 0.000 | 0.049 |  |  |  |  |  |
|  |  |  |  |  | RAM0529. Wild flesy fruits | 0.168 | 0.002 | 0.743 |  | M12. Cultivated vegetables | 0.003 | 0.000 | 0.014 |  |  |  |  |  |
|  |  |  |  |  | F01. Wild flesy fruits | 0.159 | 0.002 | 0.687 |  | M12_2. Cultivated vegetables | 0.003 | 0.000 | 0.016 |  |  |  |  |  |
|  |  |  |  |  | RT0429. Wild flesy fruits | 0.342 | 0.004 | 0.907 |  | F09. Cultivated vegetables | 0.013 | 0.000 | 0.057 |  |  |  |  |  |
|  |  |  |  |  | RT109. Wild flesy fruits | 0.329 | 0.002 | 0.879 |  | F09_2. Cultivated vegetables | 0.019 | 0.000 | 0.098 |  |  |  |  |  |
|  |  |  |  |  | RT148. Wild flesy fruits | 0.218 | 0.003 | 0.764 |  | FP01. Cultivated vegetables | 0.031 | 0.000 | 0.153 |  |  |  |  |  |
|  |  |  |  |  |  |  |  |  |  | FP01_2. Cultivated vegetables | 0.025 | 0.000 | 0.127 |  |  |  |  |  |
|  |  |  |  |  |  |  |  |  |  | HS028_2. Cultivated vegetables | 0.022 | 0.000 | 0.107 |  |  |  |  |  |
|  |  |  |  |  |  |  |  |  |  | HS0853. Cultivated vegetables | 0.003 | 0.000 | 0.016 |  |  |  |  |  |
|  |  |  |  |  |  |  |  |  |  | HS1293. Cultivated vegetables | 0.012 | 0.000 | 0.051 |  |  |  |  |  |
|  |  |  |  |  |  |  |  |  |  | HS1293_2. Cultivated vegetables | 0.013 | 0.000 | 0.061 |  |  |  |  |  |
|  |  |  |  |  |  |  |  |  |  | HS330_2. Cultivated vegetables | 0.017 | 0.000 | 0.085 |  |  |  |  |  |
|  |  |  |  |  |  |  |  |  |  | HS338. Cultivated vegetables | 0.026 | 0.000 | 0.135 |  |  |  |  |  |
|  |  |  |  |  |  |  |  |  |  | HS338_2. Cultivated vegetables | 0.028 | 0.000 | 0.128 |  |  |  |  |  |
|  |  |  |  |  |  |  |  |  |  | HS343. Cultivated vegetables | 0.006 | 0.000 | 0.028 |  |  |  |  |  |
|  |  |  |  |  |  |  |  |  |  | HS343_2. Cultivated vegetables | 0.013 | 0.000 | 0.060 |  |  |  |  |  |
|  |  |  |  |  |  |  |  |  |  | HS349_2. Cultivated vegetables | 0.018 | 0.000 | 0.096 |  |  |  |  |  |
|  |  |  |  |  |  |  |  |  |  | HS374. Cultivated vegetables | 0.013 | 0.000 | 0.058 |  |  |  |  |  |
|  |  |  |  |  |  |  |  |  |  | HS465. Cultivated vegetables | 0.023 | 0.000 | 0.129 |  |  |  |  |  |
|  |  |  |  |  |  |  |  |  |  | RAM011_2. Cultivated vegetables | 0.017 | 0.000 | 0.084 |  |  |  |  |  |
|  |  |  |  |  |  |  |  |  |  | RAM0529_2. Cultivated vegetables | 0.020 | 0.000 | 0.099 |  |  |  |  |  |
|  |  |  |  |  |  |  |  |  |  | F01_2. Cultivated vegetables | 0.027 | 0.000 | 0.149 |  |  |  |  |  |
|  |  |  |  |  |  |  |  |  |  | RT0429_2. Cultivated vegetables | 0.014 | 0.000 | 0.070 |  |  |  |  |  |
|  |  |  |  |  |  |  |  |  |  | RT109_2. Cultivated vegetables | 0.011 | 0.000 | 0.051 |  |  |  |  |  |
|  |  |  |  |  |  |  |  |  |  | RT148. Cultivated vegetables | 0.021 | 0.000 | 0.100 |  |  |  |  |  |
|  |  |  |  |  |  |  |  |  |  | M09_2. Cultivated vegetables | 0.019 | 0.000 | 0.092 |  |  |  |  |  |
|  |  |  |  |  |  |  |  |  |  | F13. Cultivated vegetables | 0.022 | 0.000 | 0.106 |  |  |  |  |  |
|  |  |  |  |  |  |  |  |  |  | F13_2. Cultivated vegetables | 0.016 | 0.000 | 0.082 |  |  |  |  |  |
|  |  |  |  |  |  |  |  |  |  | F07. Cultivated vegetables | 0.019 | 0.000 | 0.087 |  |  |  |  |  |
|  |  |  |  |  |  |  |  |  |  | F07_2. Cultivated vegetables | 0.015 | 0.000 | 0.076 |  |  |  |  |  |
|  |  |  |  |  |  |  |  |  |  | F03. Cultivated vegetables | 0.004 | 0.000 | 0.019 |  |  |  |  |  |
|  |  |  |  |  |  |  |  |  |  | M13_2. Cultivated vegetables | 0.031 | 0.000 | 0.166 |  |  |  |  |  |
|  |  |  |  |  |  |  |  |  |  | 89ACC03a_2. Wild flesy fruits | 0.458 | 0.036 | 0.886 |  |  |  |  |  |
|  |  |  |  |  |  |  |  |  |  | 89ACC04_2. Wild flesy fruits | 0.434 | 0.029 | 0.863 |  |  |  |  |  |
|  |  |  |  |  |  |  |  |  |  | ACC079_2. Wild flesy fruits | 0.521 | 0.042 | 0.925 |  |  |  |  |  |
|  |  |  |  |  |  |  |  |  |  | F05_2. Wild flesy fruits | 0.560 | 0.041 | 0.946 |  |  |  |  |  |
|  |  |  |  |  |  |  |  |  |  | M12. Wild flesy fruits | 0.877 | 0.109 | 0.992 |  |  |  |  |  |
|  |  |  |  |  |  |  |  |  |  | M12_2. Wild flesy fruits | 0.846 | 0.044 | 0.992 |  |  |  |  |  |
|  |  |  |  |  |  |  |  |  |  | F09. Wild flesy fruits | 0.499 | 0.040 | 0.900 |  |  |  |  |  |
|  |  |  |  |  |  |  |  |  |  | F09_2. Wild flesy fruits | 0.454 | 0.044 | 0.875 |  |  |  |  |  |
|  |  |  |  |  |  |  |  |  |  | FP01. Wild flesy fruits | 0.339 | 0.032 | 0.712 |  |  |  |  |  |
|  |  |  |  |  |  |  |  |  |  | FP01_2. Wild flesy fruits | 0.339 | 0.021 | 0.774 |  |  |  |  |  |
|  |  |  |  |  |  |  |  |  |  | HS028_2. Wild flesy fruits | 0.421 | 0.035 | 0.842 |  |  |  |  |  |
|  |  |  |  |  |  |  |  |  |  | HS0853. Wild flesy fruits | 0.863 | 0.145 | 0.991 |  |  |  |  |  |
|  |  |  |  |  |  |  |  |  |  | HS1293. Wild flesy fruits | 0.494 | 0.058 | 0.885 |  |  |  |  |  |
|  |  |  |  |  |  |  |  |  |  | HS1293_2. Wild flesy fruits | 0.482 | 0.050 | 0.889 |  |  |  |  |  |
|  |  |  |  |  |  |  |  |  |  | HS330_2. Wild flesy fruits | 0.464 | 0.035 | 0.892 |  |  |  |  |  |
|  |  |  |  |  |  |  |  |  |  | HS338. Wild flesy fruits | 0.417 | 0.023 | 0.863 |  |  |  |  |  |
|  |  |  |  |  |  |  |  |  |  | HS338_2. Wild flesy fruits | 0.382 | 0.034 | 0.786 |  |  |  |  |  |
|  |  |  |  |  |  |  |  |  |  | HS343. Wild flesy fruits | 0.681 | 0.079 | 0.970 |  |  |  |  |  |
|  |  |  |  |  |  |  |  |  |  | HS343_2. Wild flesy fruits | 0.516 | 0.043 | 0.908 |  |  |  |  |  |
|  |  |  |  |  |  |  |  |  |  | HS349_2. Wild flesy fruits | 0.474 | 0.026 | 0.897 |  |  |  |  |  |
|  |  |  |  |  |  |  |  |  |  | HS374. Wild flesy fruits | 0.526 | 0.051 | 0.916 |  |  |  |  |  |
|  |  |  |  |  |  |  |  |  |  | HS465. Wild flesy fruits | 0.432 | 0.016 | 0.881 |  |  |  |  |  |
|  |  |  |  |  |  |  |  |  |  | RAM011_2. Wild flesy fruits | 0.475 | 0.034 | 0.900 |  |  |  |  |  |
|  |  |  |  |  |  |  |  |  |  | RAM0529_2. Wild flesy fruits | 0.454 | 0.030 | 0.875 |  |  |  |  |  |
|  |  |  |  |  |  |  |  |  |  | F01_2. Wild flesy fruits | 0.430 | 0.023 | 0.875 |  |  |  |  |  |
|  |  |  |  |  |  |  |  |  |  | RT0429_2. Wild flesy fruits | 0.496 | 0.039 | 0.917 |  |  |  |  |  |
|  |  |  |  |  |  |  |  |  |  | RT109_2. Wild flesy fruits | 0.531 | 0.058 | 0.913 |  |  |  |  |  |
|  |  |  |  |  |  |  |  |  |  | RT148. Wild flesy fruits | 0.417 | 0.051 | 0.830 |  |  |  |  |  |
|  |  |  |  |  |  |  |  |  |  | M09_2. Wild flesy fruits | 0.447 | 0.042 | 0.867 |  |  |  |  |  |
|  |  |  |  |  |  |  |  |  |  | F13. Wild flesy fruits | 0.439 | 0.029 | 0.871 |  |  |  |  |  |
|  |  |  |  |  |  |  |  |  |  | F13_2. Wild flesy fruits | 0.478 | 0.042 | 0.899 |  |  |  |  |  |
|  |  |  |  |  |  |  |  |  |  | F07. Wild flesy fruits | 0.429 | 0.053 | 0.825 |  |  |  |  |  |
|  |  |  |  |  |  |  |  |  |  | F07_2. Wild flesy fruits | 0.499 | 0.044 | 0.913 |  |  |  |  |  |
|  |  |  |  |  |  |  |  |  |  | F03. Wild flesy fruits | 0.819 | 0.164 | 0.987 |  |  |  |  |  |
|  |  |  |  |  |  |  |  |  |  | M13_2. Wild flesy fruits | 0.411 | 0.031 | 0.844 |  |  |  |  |  |

**Table S3.** Mean isotopic ratios and digestible elemental concentrations for sampled Apennine bear key foods used for SIMMs.

|  |  | **δ13C (‰)** | |  | **δ15N (‰)** | |  | **Digestible elemental concentrations** | | |  | **n** |
| --- | --- | --- | --- | --- | --- | --- | --- | --- | --- | --- | --- | --- |
| **Sources** |  | **Mean** | **SD** |  | **Mean** | **SD** |  | **[C]** |  | **[N]** |  |  |
| Herbs |  | -26.7 | 0.65 |  | 0.2 | 1.5 |  | 45 |  | 6.2 |  | 59 |
| Hard masts |  | -24.2 | 0.61 |  | 1.7 | 2.3 |  | 61.9 |  | 2.3 |  | 12 |
| Wild fleshy fruits |  | -24.4 | 0.63 |  | -0.2 | 2.1 |  | 45 |  | 1.1 |  | 49 |
| Cultivated fleshy fruits |  | -23.8 | 0.8 |  | 3 | 3.1 |  | 45 |  | 1.0 |  | 23 |
| Cultivated vegetables |  | -25.1 | 0.33 |  | 11.4 | 3.1 |  | 45 |  | 6.9 |  | 4 |
| Ungulates |  | -23 | 0.69 |  | 8.2 | 0.5 |  | 51.5 |  | 15.3 |  | 7 |
| Formicidae |  | -24.5 | 0.5 |  | 5.6 | 1.1 |  | 51.5 |  | 6.9 |  | 26 |

**Figure S1.** Simulated mixing regions for bear hair sections isotopic values (black dots) and bear key-foods (mean SD, with dots and error bars) corrected for TEF in the four dietary seasons. (a) spring; (b) early summer; (c) late summer; (d) autumn. Colors indicated the probability that the mixing model can explain an individual consumer’s isotopic value (Smith et al. 2013).

Probability contours are at the 5% level (outermost contour) and at every 10% level.

s1: herbs; s2: hard mast; s3: wild fleshy fruits; s4: cultivated fleshy fruits; s5: cultivated vegetables; s6: Formicidae; s7: ungulates.

**
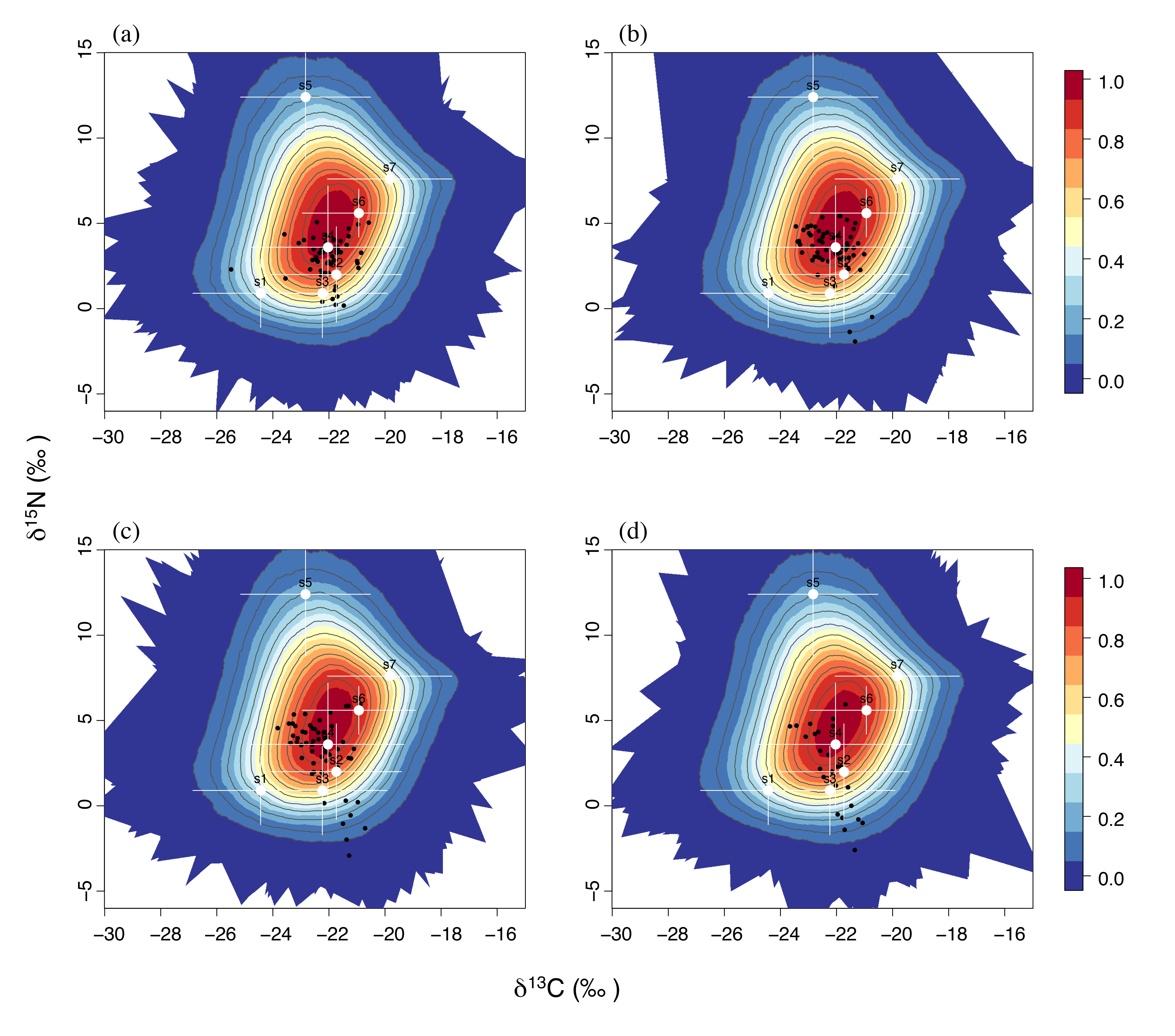
**

**Figure S2.** Mean estimated posterior proportional dietary contribution for Apennine Brown bears, obtained with uninformative priors. Circles represent the median contribution, of each key-food category, to bears diet, with 95% (thin lines) and 50% (thick lines) credibility intervals.
